# A dependent and censored first hitting-time model with compound Poisson processes

**DOI:** 10.1007/s10985-026-09705-1

**Published:** 2026-04-27

**Authors:** Mikael Escobar-Bach, Alexandre Popier, Malo Sahin

**Affiliations:** 1 LAREMA, Université d’Angers, 2 bd de Lavoisier, 49000 Angers, France; 2 Laboratoire Manceau de Mathématiques, Le Mans Université, Avenue Olivier Messiaen, 72085 Le Mans, France

**Keywords:** Survival analysis, Dependent censoring, Compound Poisson process, First hitting-time model

## Abstract

We consider a bivariate first hitting-time model in which durations are the crossing times of dependent compound Poisson processes with fixed thresholds. The identifiability of the model is discussed, and likelihood estimators of the model parameters are proposed. We obtain the asymptotic properties of the estimators and underline their finite sample performance with a simulation study on synthetic data. The practical applicability of our approach is demonstrated by an application using data from patients suffering from mushroom poisoning.

**Supplementary Information:**

The online version contains supplementary material available at 10.1007/s10985-026-09705-1.

## Introduction

The analysis of time-to-event data has always been of great interest in various applications, embedded with challenging statistical analysis when subject to censoring. First hitting-time (FHT) or threshold crossing models are attractive survival modeling approaches that allow censoring mechanisms to be described by stochastic processes. The construction of FHT models typically relies on a latent or unobservable continuous-time random process $L=(L_t,\,t\ge 0)$ and an event *B*. The event time of interest is then given by

1 $$\begin{aligned} \tau _B=\inf \{t\ge 0,\,L_t\in B\} \end{aligned}$$

referring to the first time a stop rule is met. In particular, scenarios with crossing rules consider real thresholds $x\in \mathbb {R}$ where events $B=B_x$ have the forms given by $(-\infty ,x]$ or $[x,+\infty )$ depending on whether they are up- or down-crossings. For multivariate analysis, several random times can be considered, each associated with different reaching rules. However, in the context of censored or competing events, only the smallest time value is returned, leaving the others unknown. Thus, model identification is usually ensured at the cost of independence between random times, which is referred to as independent censoring. In fact, the initial studies in Tsiatis (1975) emphasize that experiments with dependent censoring certainly prohibit statistical identifiability of the model and thus lead to drastically biased estimation. Recent work has therefore been concerned with this problem, where preliminary work can be found in Zheng and Klein (1995) with the introduction of a copula-graphic estimator based on a known copula function. The latter has been further explored in Rivest and Wells (2001) by considering Archimedean copulas and then extended to several regression models with known copula/Archimedean generator in Braekers and Veraverbeke (2005); Chen (2010); de Uña and Veraverbeke (2017); Sujica and Van Keilegom (2018); Emura and Chen (2018) among others. By definition, a copula is a joint multivariate distribution function with uniform margins that gives a margin-free characterization of the dependence structure, and whose existence has been universally proved in Sklar (1959). Some other recent developments have managed to relax the assumption of the known copula by considering parametric models in Czado and Van Keilegom (2023) and semiparametric models in Deresa and Keilegom (2024). In practice, copulas are convenient tools since they cover all possible dependence structures. However, it can be challenging for investigators without a strong mathematical background to grasp the formalism of copulas and link them effectively to the data source. Furthermore, most methods with parametric copula families rely on strong regularity conditions, such as absolute continuity or differentiability, and neglect those with singular parts, although they represent a large fraction of general copulas (Durante et al. 2015). As such, FHT models allow us to consider alternative characterizations that internalize the description of the dependence structures through stochastic constructions instead of model fitting. In survival analysis, this point of view presents substantial interest due to its realism and applicability (Lee and Whitmore 2006). For examples, the analyses in Davarzani et al. (2015, 2019); Escobar-Bach and Helali (2024) use common shock models for possibly equally dependent event times, and a model extension referred to as mixed hitting-time model, considers random levels for the analysis of optimal stopping decisions by heterogeneous agents (Abbring 2012).

In real world applications, FHT models have a long history in medicine, economics, and engineering sciences. For example, the analysis in Longini et al. (1989) has proposed a survival study on the follow-up to death for patients with human immunodeficiency virus (HIV) where the various stages of infection are based on a homogeneous time Markov process. Another study in Zenga et al. (2013) compares the length of time of Italian students at the university with those of European students. The students leaving the study prematurely are considered as censored and the length time of each of them is simulated throughout a hidden Markov chain, sometimes referred to as phase-type transition models. Other recent works have successfully used FHT models in biology, such as in Hobolth et al. (2019) with DNA modeling or in life insurance in Asmussen et al. (2019). From a heuristic approach, a study in De Bin and Stikbakke (2023) has recently proposed a boosting gradient type algorithm to fit phase-type distributions with high-dimensional covariates. Extensions of this model have also been considered with for instance crossing rules at random levels. Such construction is typically referred to as mixed hitting-time models and have examples in economics, with real applications such as patent races (Reinganum 1982; Choi 1991) and technology adoption (Jensen 1982; Farzin et al. 1998).

In this paper we propose a bivariate FHT model with compound Poisson processes. The dynamic of the model is based on the competition between two monotone processes to reach fixed levels. In line with the idea of longitudinal analysis, we assume that events are checked for completion at the same random time intervals. This means that the model can return the same event times when the processes cross their respective levels simultaneously. Such interdependency between the processes is crucial and plays a central role in identifying the model structures. Like competing models with common shocks, it allows consideration of complex follow-up experiments with two dependent competing events, even when deterministic or continuous recording is not feasible. It also extends to applications with a different scope than copula-based models with standard statistical assumptions, which are not usually suited to copulas with singular components. Our estimation uses a maximum likelihood approach applied to the parameters of the jump size distributions and the inter-arrival time intensity. The identification and consistency analyses are addressed with properties from the frameworks of renewal processes and convolution of densities. In particular, our study discusses the necessary settings and conditions on the jump size distributions to ensure the model identifiability. Answering such questions has already been the subject of several contributions with observed Lévy processes (Comte et al. 2015; Coca 2018; Duval and Mariucci 2021), and is a even more challenging in our study due to the censoring mechanism and the bivariate setting. In particular, the fixed-threshold setting turns out to be more complex to handle than the random-threshold setting, since the level heterogeneity helps to select the correct jump size distributions. Under this context, we prove the asymptotic normality of the proposed estimators under mild conditions. The finite sample performance of our approach is discussed with a study on synthetic data and its practical application is underlined with a study on mushroom poisoning.

A synopsis of our theoretical and practical contributions is outlined below:

- A thorough investigation of the model identifiability according to the censoring information is presented. The required censoring models are discussed, along with a large study of jump size distributions that allows identification of the latent stochastic processes.
- This analysis allows us to derive a maximum likelihood estimator for the process parameters. Under mild conditions, we also demonstrate the consistency of our estimator and guarantee its asymptotic normality.
- We propose a thorough experimental study involving analyses of the estimation efficiency in different scenarios using artificial data. We also emphasize the practical applicability of our model with an original application of time-to-severe-condition for patients suffering from mushroom poisoning.

The remainder of the paper is given as follows. In Sect. 2 we present the stochastic construction associated with the event times and discuss of conditions on our model representation in the context of survival analysis. Section 3 proposes an analysis of the model functions, highlighting remarkable properties of the hitting times, in perspective with the internal dependence structure of the bivariate stochastic process. In Sect. 4, we propose a parameterized configuration and discuss conditions that ensure its identifiability. A maximum likelihood approach is proposed along with asymptotic results in Sect. 5. In Sect. 6, the finite sample performance of our approach is discussed on generic data and highlight its practical applicability with an original study on poisoning data. All proofs and technical lemmas are postponed to the supplementary material.

## Model and settings

As indicated in the introduction section, our interest is in understanding the distributions of random crossing times as in (1) for a bivariate Lévy process $L=(L_1,L_2)$ and fixed thresholds. In our study, we consider two pure jumps Lévy processes whose respective jump times occur simultaneously and the jump sizes are independent. Dependence within the latent process *L* hence results from the common indexation of the marginal processes with the same Poisson process *N*, which characterizes the number of jumps at time *t*.

Formally, we denote $(E_n)_{n \in \mathbb {N}^*}$ as an i.i.d. sequence of exponential laws with intensity parameter $\lambda >0$ and define

$$\begin{aligned} N_t:= \sup \{ n \in \mathbb {N}^*, E_1 + \ldots + E_n \le t \},\quad t>0 \end{aligned}$$

where the supremum of the empty set is set to 0. The jump sizes of the processes $L_1$ and $L_2$ are respectively regrouped in the i.i.d. sequences $(X_n)_{n \in \mathbb {N}^*}$ and $(Z_n)_{n \in \mathbb {N}^*}$. All the aforementioned random series are assumed independent and define the compound Poisson processes:

$$\begin{aligned} L_{t,1} = \sum _{n=1}^{N_t} X_n = \sum _{n=1}^{+ \infty } \ X_n 1\!\!1_{ \{ E_1 + \ldots + E_n \le t \} } \quad \quad \forall t \ge 0, \\ L_{t,2} = \sum _{n=1}^{N_t} Z_n = \sum _{n=1}^{+ \infty } \ Z_n 1\!\!1_{ \{ E_1 + \ldots + E_n \le t \} } \quad \quad \forall t \ge 0. \end{aligned}$$

In this study, we assume that the marginal processes are almost surely monotonic and consider jump sizes with constant signs. Without loss of generality and to ease the forthcoming analysis, we consider the case where both are almost surely non-decreasing. The model is defined with $\mathbb {P}[Z_1=0],\mathbb {P}[X_1=0]\in [0,1)$. The possible positivity of both terms requires attention and will be important during the identifiability stage. For the sake of rigor in the forthcoming calculations, let $X_0$ and $Z_0$ be such that $X_0 = Z_0 = 0$ a.s.

For any fixed thresholds $x,z>0$, we define the up-crossing times of the processes $L_1$ and $L_2$ by

$$\begin{aligned} T\; = \; \inf \{ t \ge 0; \quad L_{t,1} \ge x \}\quad \text {and}\quad C \; = \; \inf \{ t \ge 0; \quad L_{t,2} \ge z \}. \end{aligned}$$

In our context, the process *L* is unobservable so that the distributions of the event times *T* and *C* are the only statistical information available. In addition, due to the censoring mechanism, only the smallest random time is returned, in a sense that both processes $L_1$ and $L_2$ compete to be the first to cross their respective thresholds. In survival analysis, this setting is characterized by pairwise outcomes that return the uncensored random time and an indicator of the observed time. Related to this framework, we consider two types of survival models $(Y,\Delta )$, depending on the censoring information available.

$$\begin{aligned} {\textbf {Model I:}} \quad \quad \quad&(Y, \Delta ) \; = \; ( \min \{ T ; C \} , 1\!\!1_{ \{T \le C \} } ) \\ {\textbf {Model II:}} \quad \quad \quad&(Y, \Delta ) \; = \; ( \min \{ T ; C \} , 0. 1\!\!1_{\{ T > C \}} + 1 . 1\!\!1_{\{ T < C \}} + 2 . 1\!\!1_{\{ T = C \}} ) \end{aligned}$$

Scenarios drawn from Model I are usually studied with independent censoring while Model II can be seen as a competing risk configuration. The analysis in Escobar-Bach and Helali (2024), where dependent censoring occurs and $\mathbb {P}[ T = C ] > 0$, has shown that non-parametric identifiability is not always guaranteed considering only Model I as opposed to Model II. Beyond the applicability of Model II in some experimental contexts, these settings will be discussed to address the model identifiability in Sect. 4. From a statistical perspective, we will work under a parametric framework for the inter-arrival times of the process *L* and the jump size distributions. Depending on the class of the model and the definition of the outcomes, our objective is to propose an estimation procedure of the model parameters that fully describes the law of *L*. This will then make explicit the distribution of event times *T* and *C*, which are usually of primary interest in survival analysis.

*Statistical context* In our problem, $X_1$ and $Z_1$ are supposed to belong in some parametrized families of probability laws. In our context, we observe realizations of the censoring couple $(Y, \Delta )$ from Model I or II, depending on which class of model we lie in. The usual objective of a survival model with censoring is to determinate the respective laws of the variable of interest T and of the censoring variable C. Our objective is to build an estimator of the vector of parameters that fully describe the law of *L*, naturally leading to the derivation of the laws of *T* and *C*. The article (White 1982) gives explicit assumptions to be verified on the parametrized density function $f_{(Y,\Delta )}$ to obtain respectively the consistency and the asymptotic normality of the Maximum likelihood estimator.

## Theoretical analysis of model functions

In the following section, we propose a prior analysis of the representations and the properties of the model functions. The structure of the model induces a dependency between the two hitting times due to the fact that the completion of the crossing rules can occur simultaneously with nonzero probability. This particularly implies that the couple (*T*, *C*) dependence structure admits a singular part supported on the set $\{(u,v)\in \mathbb {R}^2_+,\,u=v\}$. We derive the couple density function in the following lemma and illustrate this property in Remark 3.3.

### Lemma 3.1

(Crossing time density function) The absolutely continuous part of the law of the couple (*T*, *C*) is supported by the set $\{(u,v) \in (\mathbb {R}_+)^2, \ u \ne v\}$ and has a density function given by

2 $$\begin{aligned}&f_{ac}(u,v) \nonumber \\&= \sum _{n=1}^{+ \infty } \sum _{j=1}^{n} \big [ c_{j-1,X} - c_{j,X} \big ] \big [ c_{n,Z} - c_{n+1,Z} \big ] \lambda ^2 e^{ - \lambda v} \frac{ (\lambda (v - u))^{n-j} }{ (n-j)! } \frac{( \lambda u )^{j-1}}{(j-1)!} 1\!\!1_{ \{ u < v \} } \end{aligned}$$

3 $$\begin{aligned} + \sum _{n=1}^{+ \infty }&\sum _{j=1}^{n} \big [ c_{n,X} - c_{n+1,X} \big ] \big [ c_{j-1,Z} - c_{j,Z} \big ] \lambda ^2 e^{ - \lambda u} \frac{ (\lambda (u - v))^{n-j} }{ (n-j)! } \frac{( \lambda v )^{j-1}}{(j-1)!} 1\!\!1_{ \{ u > v \} }. \end{aligned}$$

The singular part is supported by the diagonal $\{(u,u), u \ge 0\}$ with a density function given by

4 $$\begin{aligned} f_s(u)= \partial _u \mathbb {P}[T\le u, T=C] \; = \; \sum _{n=0}^{+ \infty }&\big [ c_{n,X} - c_{n+1,X} \big ] \big [ c_{n,Z} - c_{n+1,Z} \big ] \lambda e^{ - \lambda u } \frac{(\lambda u)^n}{n!} , \end{aligned}$$

where in both formulas for any $n \in \mathbb {N}$

$$\begin{aligned} c_{n,X} = \mathbb {P} \left( \sum _{i=0}^n X_i< x \right) \quad \quad \quad \text {and} \quad \quad \quad c_{n,Z} = \mathbb {P} \left( \sum _{i=0}^n Z_i < z \right) . \end{aligned}$$

This singular property due to the coupling of the crossing times plays a central role in our model dynamics. In a sense, one can think of an external agent returning the times at which the crossing rules have been observed. This leaves open the possibility that the events share the same time window, which results in a common returned time from the agent. The following lemma gives an explicit formula of the probability that such event occurs in terms of the jump sizes distributions.

### Lemma 3.2

The probability that *T* and *C* occur at the same time is given by

5 $$\begin{aligned} \mathbb {P} \left[ T = C \right] = \underset{n \in \mathbb {{N}}}{\sum } [c_{n,X} - c_{n+1,X} ] [c_{n,Z} - c_{n+1,Z} ] . \end{aligned}$$

Moreover, the previous probability is null if and only if one of the crossing times occurs almost surely before the other one, i.e. we have $\mathbb {P} \left[ T = C \right] = 0$ if and only if $T<C$ almost surely or $C<T$ almost surely.

### Remark 3.3

It is worth noting that the probability of equaled event times does not depend on the intensity parameter $\lambda$. This can be explained intuitively, since the simultaneous occurrence depends only on the number of jumps between the processes $L_1$ and $L_2$ and not on the jump times.

As an immediate consequence of Lemma 3.2, we observe that the model is non trivial if the probability that *T* and *C* occurs at the same time is strictly positive. Indeed, configurations under the null probability automatically induce either almost certainly censored or uncensored data, which leads to the non-identifiability of the law of *T* or *C*. In the sequel, we will thus only consider processes such that $\mathbb {P} \left[ T=C \right] >0$. In the next lemma, we derive the density of the censoring couple under models I and II.

### Lemma 3.4

(Survival outcome density function) The density function of the couple $(Y,\Delta )$ is given by

**Model I:**

$$\begin{aligned} f_{(Y, \Delta )} (t, \delta ) =&\left\{ \begin{matrix} \sum _{n=0}^{+ \infty } [c_{n,X} - c_{n+1,X} ] c_{n,Z} \lambda e^{- \lambda t} \frac{(\lambda t)^n}{n!} 1\!\!1_{ \{ t \ge 0 \} } & \quad \text {if} \; \delta = 1 \\ \sum _{n=0}^{+ \infty } [c_{n,Z} - c_{n+1,Z} ] c_{n+1,X} \lambda e^{- \lambda t} \frac{(\lambda t)^n}{n!} 1\!\!1_{ \{ t \ge 0 \} } & \quad \text {if} \; \delta = 0 \end{matrix} \right. \\ \end{aligned}$$

**Model II:**

$$\begin{aligned} f_{(Y, \Delta )} (t, \delta ) =&\left\{ \begin{matrix} \sum _{n=0}^{+ \infty } [c_{n,Z} - c_{n+1,Z} ] c_{n+1,X} \lambda e^{- \lambda t} \frac{(\lambda t)^n}{n!} 1\!\!1_{ \{ t \ge 0 \} } & \text {if} \; \delta = 0 \\ \sum _{n=0}^{+ \infty } [c_{n,X} - c_{n+1,X} ] c_{n+1,Z} \lambda e^{- \lambda t} \frac{(\lambda t)^n}{n!} 1\!\!1_{ \{ t \ge 0 \} } & \text {if} \; \delta = 1 \\ \sum _{n=0}^{+ \infty } [c_{n,X} - c_{n+1,X} ] [c_{n,Z} - c_{n+1,Z} ] \lambda e^{- \lambda t} \frac{(\lambda t)^n}{n!} 1\!\!1_{ \{ t \ge 0 \} } & \text {if} \; \delta = 2 \end{matrix} \right. \end{aligned}$$

In the sequel, a maximum likelihood approach is proposed in section 5 in which density functions are computed numerically according to the above derivations. Due to practical limitations, we have to consider finite series when approximating the model functions. The following lemma provides a uniform bound for the aforementioned series, which allows one to control the theoretical discrepancy between $f_{(Y, \Delta )}$ and its numerical approximation.

### Lemma 3.5

Let $\lambda _m>0$ be an arbitrary constant such that $\lambda \le \lambda _m$. For any $N \in \mathbb {N}$, define $f_{N,(Y, \Delta )}$ the partial sum up to *N* of $f_{(Y, \Delta )}$. Then for any $\tau >0$, we have

$$\begin{aligned} \log \left( f_{(Y, \Delta )}(t,\delta ) \right) =&\log \left( f_{N,(Y, \Delta )} (t,\delta )\right) + r_N(t,\delta ) \end{aligned}$$

such that $$\forall (t,\delta ) \in [0,\tau ] \times \{0,1,2\}$$,

$$\begin{aligned} 0\le r_N(t, \delta )&\le \min _{j \in \{0, \ldots , N \}} \left\{ \frac{j !}{(\lambda _m \tau )^{j}} b_{j,N} \left( \sum _{n\ge N+1} \dfrac{(\lambda _m\tau )^n}{n!} \right) \right\} \\&\le \min _{j \in \{0, \ldots , N \}} \left\{ \frac{j !}{(\lambda _m \tau )^{j}} b_{j,N} e^{\lambda _m \tau } \right\} \end{aligned}$$

where $$b_{j,N} = \max \left\{ \frac{\sup _{n> N} \{ (c_{n,X} - c_{n+1,X}) c_{n,Z} \}}{\left( c_{j,X} - c_{j+1,X} \right) c_{j,Z}}, \frac{\sup _{n > N} \{ (c_{n,Z} - c_{n+1,Z}) c_{n+1,X} \}}{\left( c_{j,Z} - c_{j+1,Z} \right) c_{j+1,X}}\right\} $$.

The previous lemma helps practitioners to select an appropriate order of the *N* value to obtain the desired level of precision. Note that when accurate bounds or exact expressions are provided for the sequences $(c_{n,X})_{n \in \mathbb {N}}$ and $(c_{n,Z})_{n \in \mathbb {N}}$, the index *j* that achieves the minimum value in the above inequality can be easily found in terms of the values of $\lambda _m\tau$. Furthermore, Theorem 4.6 will also provide accurate bounds for the sequences $(c_{n,X})_{n \in \mathbb {N}}$ and $(c_{n,Z})_{n \in \mathbb {N}}$, enabling a more precise approximation according to the selected jump distributions.

In the next section, analytical properties of the hazard function of *Y* will help to identify the intensity parameter $\lambda$. The next lemma proposes a representation of the hazard function that observes proportionality with the latter parameter.

### Lemma 3.6

The hazard function of the random variable *Y* is given by

6 $$\begin{aligned} h_{Y}(t) = \frac{f_{Y}(t)}{S_{Y}(t)} = \lambda \left( 1- \dfrac{ \sum _{n=0}^{+ \infty } c_{n+1,X} c_{n+1,Z} \frac{(\lambda t)^n}{n!} }{ \sum _{n=0}^{+ \infty } c_{n,X} c_{n,Z} \frac{(\lambda t)^n}{n!} }\right) \end{aligned}$$

where $S_{Y}$ denotes the survival function of *Y*.

## Identifiability

### Notation

In line with our statistical purposes, we consider a parametric framework and assume that the jump size laws belong to pre-specified parametric distribution classes. To reflect this setting in the rest of the paper, we propose to index the previous notations with new parameters. We thus assume that the distributions of $\mathbb {P}_{X_1}$ and $\mathbb {P}_{Z_1}$ respectively belong to the classes $\{ \mathbb {P}_{\alpha }, \alpha \in \Theta _1 \}$ and $\{ \mathbb {P}_{\beta }, \beta \in \Theta _2 \}$ where $\Theta _i$, $i=1,2$ stand as real parameter spaces. We also denote the full parameter space as $\Theta =\Lambda \times \Theta _1 \times \Theta _2$ and index any model functions or probabilities with $\theta =(\lambda ,\alpha ,\beta )\in \Theta$ if there is a dependence on the jump size distributions or the counting process intensity. In particular, the coefficients of the cumulative jumps are now denoted by

$$\begin{aligned} c_{n,X}(\alpha ) = \mathbb {P}_{\alpha } \left( \sum _{i=0}^n X_i< x \right) \quad \quad \quad \text {and} \quad \quad \quad c_{n,Z}(\beta ) = \mathbb {P}_{\beta } \left( \sum _{i=0}^n Z_i < z \right) . \end{aligned}$$

*Identification in survival analysis* It is important to mention that the issue of identifiability is strongly intertwined with the dependency between event times. Unlike independent censoring, which allows non-parametric identification, the complex dependence structure resulting from our model requires much more sophisticated analysis. In particular, its singular part contradicts requirements for some well defined approaches such as in Braekers and Veraverbeke (2005) with the copula-graphic estimator and in Czado and Van Keilegom (2023) with a parametric setting. In the survival analysis framework, model identifiability has to take into account the censoring issue with an alternate definition than in standard scenarios. Formally, for any two parameter values $\theta ,\theta ' \in \Theta$, we say that the survival model is identifiable if

7 $$\begin{aligned} f_{(Y, \Delta ),\theta } = f_{(Y, \Delta ), \theta '} \quad \Longrightarrow \quad \theta = \theta '. \end{aligned}$$

However, criteria only based on the density function of the observed data does not allow to immediately obtain the above property. This difficulty is particularly illustrated in the following counter-example based on the exponential distribution.

### Counter-example 4.1

Consider two intensity parameters $\lambda ,\lambda '>0$ and two sequences $(b_n)_{n\in \mathbb {N}}$, $(b'_n)_{n\in \mathbb {N}}$ with $\lambda = 1$, $\lambda ' = 2/3$, $b_n= (1/2)^n$ and $b_n' = (1/4)^n$. Then for any $t\ge 0$, one we have

$$\begin{aligned} e^{- \lambda t} \sum _{n=0}^{+ \infty } b_n \frac{(\lambda t)^n}{n!} = e^{- \lambda ' t} \sum _{n=0}^{+ \infty } b'_n \frac{(\lambda ' t)^n}{n!}. \end{aligned}$$

The previous example thus shows that fine-tuning parameters can result in equal density functions of $(Y,\Delta )$, even though they have different values. Nonetheless, we can prove that identification of the intensity parameter and cumulative jump coefficients are possible under different configurations of the parametric classes.

### Definition 4.2

Our model complies with the assumption $I_n$, if for any parameter values $\theta \in \Theta$ and $\theta '\in \Theta$

$$\begin{aligned} f_{(Y, \Delta ),\theta } = f_{(Y, \Delta ), \theta '}\Longrightarrow c_{k,X}(\alpha )=c_{k,X}(\alpha ')\text { and } c_{k,Z}(\beta )=c_{k,Z}(\beta '),\forall k\le n, \end{aligned}$$

where by convention, satisfying the hypothesis $I_n$ for any $n\in \mathbb {N}$ is written $I_\infty$.

It is important to emphasize that fulfillment of the $I_n$ assumption does not necessarily ensure survival identifiability. From a stochastic process perspective, this also represents a complicated problem, which can be seen as the difficulty to characterize renewal processes only from the resulting crossing time distributions. Theoretical arguments such as the absence of stationary increments for renewal processes justify the difficulty of this question. Contrarily to the particular case of Poisson processes, most of the renewal processes are not Lévy processes (see Lemma 1.1 from Kosto and Mitov (2014)). However, the analysis of our model with several configurations has shown that almost all standard parametric classes under $I_n$ assumption have identifiable survival models. This suggests that reasonable settings with our model description mostly return practical survival models with likelihood methods. This property is discussed at the end of this section where we propose several assumptions to guarantee $I_n$ or $I_\infty$ under mild conditions.

*Model identifiability* Our identification procedure is twofold: first, a prior analysis of the hazard function of *Y* shows that our survival model allows identification of the intensity parameter, and then we show that the assumption $I_n$ is verified for some $n \in \mathbb {N}$.

Derivation of the hazard function in Lemma 3.6 allows to isolate the intensity parameter in such way that the function can be written as $\lambda (1-g)$ where one can determine *g*’s limit at infinity. In the following lemma, we show how this form is based on the cumulative jump size sequences.

### Lemma 4.3

Let $(a_n)_{n \in \mathbb {N}}$ and $(b_n)_{n \in \mathbb {N}}$ be two sequences of positive real numbers. Let *g* be the function defined on $\mathbb {R}^*_+$ by

$$\begin{aligned} g(t) = \frac{\sum _{n=0}^{+ \infty } a_{n+1} b_n t^n}{ \sum _{n=0}^{+ \infty } a_{n} b_n t^n }, \quad t > 0. \end{aligned}$$

Suppose that there exists $a \in [0,1]$ such that $\lim _{n \xrightarrow {} + \infty } \frac{a_{n+1}}{a_n} = a$, then $\lim _{t \xrightarrow {} + \infty } g(t) = a$.

Lemma 4.3 suggests that knowledge of the limit *a* of $(c_{n+1,X}c_{n+1,Z})/(c_{n,X} c_{n,Z})$ can help isolating the intensity parameter since the limit of the hazard function at infinity gives $\lambda (1-a)$. As such, it is important to determine under which conditions one can determine the value of *a*.

In order to answer that question, we define the three following families of random variable sequences.

### Definition 4.4

Let $M = (M_n)_{n \in \mathbb {N}^*}$ be a sequence of i.i.d. non-negative random variables.

Class $F_1$.: $M_1$ is almost surely greater than a positive constant.
Class $F_2$.: $M_1$ is absolutely continuous with respect to the Lebesgue measure and the density denoted by $\phi$ satisfies

- For all $$\varepsilon > 0$$, $$\mathbb {P}(M_1 \in [0, \varepsilon ] ) = \int _0^\varepsilon \phi > 0$$.
- There exists $N > 0$ such that $\phi ^{*N} = \phi * \ldots * \phi$ is non-decreasing on the subset [0, *x*].

Note that $\phi ^{*N}$ is the density function of the sum $S_N = \sum _{k=1}^{N} M_k$.

Class $F_3$.: $M_1$ is a discrete random variable, $0 \in M_1(\Omega )$, $\inf ( M_1(\Omega )\backslash \{ 0 \}) \in M_1(\Omega )$ and $\inf ( M_1(\Omega )\backslash \{ 0 \}) > 0$.

### Remark 4.5

The existence of $N > 0$ such that $\phi ^{*N}=\phi _{S_N}$ is non-decreasing on the subset [0, *x*] is a property that can be verified in practice. Indeed, the function $\phi ^{*N}$ is determinable for lot of density functions. Easiest cases are the infinitely divisible and stable laws. For example the class of gamma distributions, uniform laws on the compact subset [0, *a*], with $a >0$, Fréchet distributions, Lévy distributions, etc. belong to $F_2$.

In the sequel, we consider that $(X_n)_{n \in \mathbb {N}}$ and $(Z_n)_{n \in \mathbb {N}}$ respectively belong to the classes $F_1$, $F_2$ or $F_3$. If $(X_n)_{n \in \mathbb {N}}$ (resp. $(Z_n)_{n \in \mathbb {N}}$) belongs to the family $A_1$, there exists $N \in \mathbb {N}$ such that for any $n \ge N$, $c_{n,X} = 0$ (resp. $c_{n,Z}=0$). The following theorem determines the limit at infinity of the sequences $\left( c_{n+1,X} /c_{n,X} \right) _{n \in \mathbb {N}}$ and $\left( c_{n+1,Z} / c_{n,Z} \right) _{n \in \mathbb {N}}$, depending on which family of probability laws they belong to $F_1$ or $F_2$.

### Theorem 4.6

If $(X_n)_{n \in \mathbb {N}^*} \in F_2$. Then we have

$$\begin{aligned} \lim _{n \xrightarrow {} + \infty } \frac{\mathbb {P} [ X_1 + \ldots + X_{n+1}< x ]}{\mathbb {P} [ X_1 + \ldots + X_n < x]} = 0. \end{aligned}$$

If $(X_n)_{n \in \mathbb {N}^*} \in F_3$. Then, for any $x>0$, there exists $C_x > 0$ such that

$$\begin{aligned} \left| \frac{\mathbb {P} [ X_1 + \ldots + X_{n+1}< x] }{ \mathbb {P} [ X_{1} + \ldots + X_n < x] } - \mathbb {P} [X_1 = 0] \right| \le \frac{C_x}{n}. \end{aligned}$$

The sequence $\left( c_{n+1,X} / c_{n,X} \right) _{n \in \mathbb {N}}$ admits different limits whether $(X_n)_{n \in \mathbb {N}}$ belongs to $F_2$ or $F_3$. From that observation, we understand the necessity to distinguish the identifiability problem in three different cases.

### Assumption 1

We now define the following conditions that separate these cases. In all three statements, the property is supposed true regardless of the values of the parameters.

- **(H1).** $(X_n)_{n \in \mathbb {N}}$ or $(Z_n)_{n \in \mathbb {N}}$ belong to $F_1$.
- **(H2.i).** $(X_n)_{n \in \mathbb {N}}$ or $(Z_n)_{n \in \mathbb {N}}$ belong to $F_2$.
- **(H2.ii).** $(X_n)_{n \in \mathbb {N}}$ and $(Z_n)_{n \in \mathbb {N}}$ belong to $F_3$.

Assumption **(H1)** and **(H2.i)** both ensure that $\lim _{t\rightarrow +\infty }h_{Y}(t) = \lambda$, while under assumption **(H2.ii)**, combining Lemma 4.3 and Theorem 4.6, only gives $\lambda (1 - \mathbb {P}_{\alpha } [ X_1 = 0 ] \mathbb {P}_{\beta } [ Z_1 = 0 ]) = \lambda '(1 - \mathbb {P}_{\alpha '} [ X_1 = 0 ] \mathbb {P}_{\beta '} [ Z_1 = 0 ])$ which is insufficient to obtain the intensity parameter identifiability. Model I provides less information than Model II. Thus, configurations such as under **(H2.ii)** require a more informative model to obtain the intensity parameter identification, explaining the necessity to consider Model II.

### Theorem 4.7

(Identifiability)

- Under **(H1)**, considering Model I, the identifiability of the intensity parameter $\lambda$ is ensured and $I_{N_{\max }}$ is verified, with $N_{\max } = \min \{ n \in \mathbb {N}, \mathbb {P}_{\alpha }[X_1 + \ldots + X_n< x] = 0 \text { or } \mathbb {P}_{\beta }[Z_1 + \ldots + Z_n < z] = 0 \}$.
- Under **(H2.i)**, considering Model I, the identifiability of the intensity parameter $\lambda$ is ensured and $I_{\infty }$ is verified.
- Under **(H2.ii)**, considering Model II, the identifiability of the intensity parameter $\lambda$ is ensured and $I_{\infty }$ is verified.

Theorem 4.7 is a first step to obtain the identifiability of the model in three general cases. We obtain the identifiability of the intensity parameter $\lambda$, and depending on the class of model, $I_{\infty }$ or $I_n$ is verified for some $n \in \mathbb {N}$. It is worth noting that **(H2)** ensures $I_{\infty }$ , guarantying the existence of an infinite dimensional system of equations that respectively link $\alpha$, $\alpha '$ and $\beta$, $\beta '$, which are finite dimensional vectors of parameters. Moreover, the property $I_n$ induces the identifiability of the jumping variable parameters whenever $n\in \mathbb {N}^*$ is large enough. It therefore follows that a wide variety of type I models induce the identifiability from definition (7). The following example gives the minimal number of equations needed to obtain the identifiability of the jumping parameter.

### Example 4.8

Given $I_n$, we define $N_{\min } = \min \{ n \in \mathbb {N}^*, I_n \Rightarrow \alpha = \alpha ' \}$. If $X_1 \sim \mathcal {E}(\alpha )$ and $X_1' \sim \mathcal {E}(\alpha ')$, $N_{\min } = 1$. If $X_1 \sim \mathcal {B}(p)$ and $X_1' \sim \mathcal {B}(p')$, $N_{\min } = \min \{ n \ge x\}$. If $X_1 \sim \mathcal {P}(\alpha )$ and $X_1' \sim \mathcal {P}(\alpha ')$, $N_{\min } = 1$ where $\mathcal {E}$ denotes the exponential law, $\mathcal {B}$ denotes the Bernoulli law and $\mathcal {P}$ denotes the Poisson law.

It is possible to build counter-examples such as Example 4.1, where $I_n$ does not imply the jumping parameter identification $(\alpha ,\beta )$.

### Counter-example 4.9

Assume that the model lies under assumption **(H1)**. Suppose that $Z_1 = Z_1' = 1$ a.s. and that $X_1$ and $X_1'$ belongs to the family of binomial laws $\{ \mathcal {B}(n,p), (n,p) \in \mathbb {N}^* \times ]0;1[ \}$. Finally suppose that $x=9$ and $z=3$. If $X_1 \leadsto \mathcal {B}(n,p)$ and $X_1' \leadsto \mathcal {B}(n',p')$. It comes from simple calculus that $\lambda = \lambda '$ but if $n=3$ and $n'=4$ only one equation links *p* and $p'$:

$$\begin{aligned} & \mathbb {P} [ X_1 + X_2 + X_3< x] = \mathbb {P} [ X_1' + X_2' + X_3' < x]\\\iff & 1 - p^9 = 1 - \sum _{k=9}^{12} \left( {\begin{array}{c}12\\ k\end{array}}\right) (p')^k (1-p')^{12-k}. \end{aligned}$$

And for any $p \in (0,1)$ fixed, there exists a unique $p'$ such that the the equality holds. While the initial processes are not the same (the laws of $X_1$ and $X_1'$ are not the same), they induce the same censoring density function. One can observe that in this counter-example, only one equation links (*n*, *p*) to $(n',p')$.

Although the latter example shows the possible non-identifiability of some model, one can see that it has been precisely defined to permit only one equation to link the two unknown parameters. It illustrates that non-identifiability can occur in exotic models where the number of equations that link the parameters is strictly less than the dimension of the vector of parameters. However for a great variety of examples, identifiability holds. See Example 4.8 or examples of Sect. 6.1.

## Consistency and asymptotic normality of the MLE estimator

From now on, Assumption 1 holds, that is **(H1)**, **(H2.i)** or **(H2.ii)** is verified and we suppose that the identifiability equation (7) is satisfied.

In this section, we propose an MLE approach in order to estimate the parameter of our model $\theta$. All the results exposed in this section are based on convergence results from White (1982). In his article, the author gives a list of six assumptions (denoted A1 to A6) to obtain the consistency and the asymptotic normality of the MLE estimator. Verification must be done to see if the conditions of White (1982) are verified in our setting under mild assumptions.

### Notation

Given a n-sample of i.i.d. realisations $\{ (Y_i,\Delta _i), \; i \in \{1,\ldots ,n\} \}$, induced by the vector of parameters $\theta ^0 = (\lambda ^0, \alpha ^0, \beta ^0)$, we define the log-likelihood function of the sample as

$$\begin{aligned} \theta \in \Theta \mapsto \mathcal {L}_n ((Y,\Delta ),\theta )&= \frac{1}{n} \sum _{i=1}^n \log f_{(Y,\Delta ),\theta }((Y_i,\Delta _i)) \end{aligned}$$

where $f_{(Y,\Delta ),\theta }$ is defined in Lemma 3.4 (here we emphasize the dependance w.r.t. parameter $\theta$), and we define the maximum likelihood estimator as a parameter vector $\hat{\theta }_n$

$$\begin{aligned} \hat{\theta }_n&= \arg \underset{\theta \in \Theta }{\max }\ \mathcal {L}_n ((Y,\Delta ),\theta ) \end{aligned}$$

It is worth noting that White (1982) defines an MLE estimator for the more general case of misspecified model. Our model lies in the family of well specified models, simplifying the verification of the assumptions proposed in the previous article. In order to lighten the main text, all the properties needed to ensure the existence, the consistency and the asymptotic normality of the MLE are properly defined and detailed in the mathematical appendix. To guarantee the existence of a measurable MLE, classical assumptions such as the compactness of the space of parameters **(H3)** and continuity in parameters of the density functions **(H4)** are made.

- **(H3).** $\Lambda$, $\Theta _1$ and $\Theta _2$ are respectively compact subsets of $\mathbb {R}_+^*$, $\mathbb {R}^{d_1}$ and $\mathbb {R}^{d_2}$ so that $\Theta = \Lambda \times \Theta _1 \times \Theta _2$ is a compact subset of the Euclidean space $\mathbb {R}^{1+d_1+d_2}$. And the true parameter $\theta ^0$ is in the interior of $\Theta$.
- **(H4).** See Supplementary Material, Assumption 2.

Given the structure of the density function (Lemma 3.4), the continuity in $\lambda$ is immediately deduced. Assumption **(H4)** is then settled to ensure the continuity of the respective coefficient functions $\alpha \mapsto c_{n,X} (\alpha )$ and $\beta \mapsto c_{n,Z} (\beta )$, for any $n \in \mathbb {N}$.

### Theorem 5.1

Existence) Under assumptions **(H3)**-**(H4)**, there exists a measurable MLE $\hat{\theta }_n$, for any $n \in \mathbb {N}^*$.

Although the previous theorem ensures the existence of a MLE, an assumption on its uniqueness needs to be verified. The author recall in White (1982) that well specified models admit a unique minimum to it. So that assumption A3)b) is always verified in our configuration. In order to obtain consistency, a uniform bound on the family of the log of the parametrized density functions is required. A way to ensure this property is to consider the jumping variables $X_1$ and $Z_1$ with respectively regular support with respect to the parameter $\alpha$ and $\beta$. We then assume:

- **(H5).** The support of $X_1$ (resp. $Z_1$) does not depend on $\alpha$ (resp. $\beta$).

This assumption is common with MLE approaches on parametrized family of distributions and encompasses a wide class of distributions. Only few parametric family such as uniform laws on [0, *a*], $a \in \Theta _1 \subset \mathbb {R}_+^*$, have varying support. Now that all the tools required are defined, we obtain the following theorem.

### Theorem 5.2

Assume that ${\textbf {(H3)-(H5)}}$ are verified. Considering either Model I under ${\textbf {(H1)}}$ or ${\textbf {(H2.i)}}$ or Model II under ${\textbf {(H2.i)}}$, we have for almost every sequence $((Y_i,\Delta _i))_{i \ge 1}$, $\hat{\theta }_n \underset{n \rightarrow + \infty }{\longrightarrow {}} \theta ^0$.

The asymptotic normality requires more regularity on the family of parametrized density functions. In particular, it needs to be of class $\mathcal {C}^2$ on the space of parameter in a such way that derivatives of first and second orders admit proper uniform dominating integrable function. Assumptions are required to ensure the functions $\alpha \mapsto c_{n,X} (\alpha )$ and $\beta \mapsto c_{n,Z} (\beta )$ to be of class $\mathcal {C}^2$:

- **(H6).** See details in Supplementary Material, Assumption 3.

A major difficulty is to obtain functions that uniformly dominate the respective derivatives of first and second order. The compactness of the space of parameters **(H3)** and the regularity of the support of the jumping variables **(H5)** constitute the main theoretical arguments that allow to justify the existence of such functions.

To study the asymptotic normality, when ${\textbf {(H6)}}$ is verified, we define the matrices

8 $$\begin{aligned} A_n(\theta ) =&\left( \frac{1}{n} \sum _{i=1}^n \partial ^2 \log f_{(Y,\Delta ),\theta }((Y_i,\Delta _i)) / (\partial \theta _j \partial \theta _k) \right) _{1\le j,k \le 1 + d_1 + d_2} \nonumber \\ B_n(\theta ) =&\left( \frac{1}{n} \sum _{i=1}^n \partial \log f_{(Y,\Delta ),\theta }((Y_i,\Delta _i))/ \partial \theta _j \; . \; \partial \log f_{(Y,\Delta ),\theta }((Y_i,\Delta _i))/ \partial \theta _k \right) _{1\le j,k \le 1 + d_1 + d_2} \end{aligned}$$

9 $$\begin{aligned} A(\theta ) =&\left( \mathbb {E} [ \partial ^2 \log f_{(Y,\Delta ),\theta }((Y,\Delta ))/ (\partial \theta _j \partial \theta _k) ] \right) _{1\le j,k \le 1 + d_1 + d_2} \nonumber \\ B(\theta ) =&\left( \mathbb {E} [ \partial \log f_{(Y,\Delta ),\theta }((Y,\Delta ))/ \partial \theta _j \; . \; \partial \log f_{(Y,\Delta ),\theta }((Y,\Delta ))/ \partial \theta _k ] \right) _{1\le j,k \le 1 + d_1 + d_2} . \end{aligned}$$

In our framework $A(\theta )=-B(\theta )$ (see Lemma 4 in Supplementary Material). When the appropriate inverses exist, define

$$ C_n(\theta ) = A_n(\theta )^{-1} B_n(\theta ) A_n(\theta )^{-1}, \qquad C(\theta ) = - A(\theta )^{-1}. $$

### Theorem 5.3

Assume that ${\textbf {(H3)-(H6)}}$ are verified and that $A(\theta ^0)$ is non-singular. Considering either Model I under ${\textbf {(H1)}}$ or ${\textbf {(H2.i)}}$ or Model II under ${\textbf {(H2.ii)}}$, we have the following asymptotic normality

$$\begin{aligned} \lim _{n\rightarrow +\infty } \sqrt{n} ( \hat{\theta }_n - \theta ^0) \overset{d}{=}\ \mathcal {N} ( 0, C(\theta ^0)), \end{aligned}$$

where $\mathcal {N} ( 0, C(\theta ^0))$ denotes a normal distribution of expected value 0 and covariance matrix $C(\theta ^0)$. Moreover, $C_n(\hat{\theta }_n)$ converges a.s. to $C(\theta ^0)$, element by element.

## Simulation and real data analysis

### A small simulation study

In this subsection, we illustrate the performance of the proposed MLE estimator $\theta _n = (\widehat{\lambda }_n, \widehat{\alpha }_n,\widehat{\beta }_n)$ and on the estimated distribution functions of the marginal up-crossing variables T and C. We denote $\widehat{F}_{T,\lambda ,\alpha ,n}$ and $\widehat{F}_{C,\lambda ,\beta ,n}$ those functions. We consider $M=100$ samples of sizes $n = 50,100,200$ under the following models:

- $\lambda = 1.42$, $X_1 \sim \mathcal {B}(\alpha )$, with $\alpha = 0.36$ and $Z_1 = 1$ a.s., with $(x,z) = (7,17)$,
- $\lambda = 1.42$, $X_1 \sim \mathcal {E}(\alpha )$, with $\alpha = 0.71$ and $Z_1 \sim \mathcal {E}(\beta )$, with $\alpha = 2.04$, with $(x,z) = (14,7)$,
- $\lambda = 1.42$, $X_1 \sim \mathcal {B}(\alpha )$, with $\alpha = 0.36$ and $Z_1 \sim \mathcal {P}(\beta )$, with $\beta = 1.23$, with $(x,z) = (7,19)$,

where $\mathcal {B}$, $\mathcal {E}$ and $\mathcal {P}$ respectively denote the Bernoulli, the Exponential and the Poisson distributions. The choice of those three model is made in order to illustrate all the identifiable models proposed in Sect. 4. Examples a, b and c respectively belong to (H1), (H2)i) and (H2)ii). In order to obtain a qualitative measure of the efficiency of the estimator, we drew the mean squared error denoted as *nx* and the *bias* function of the estimated marginal distributions with respect to the true distribution functions. They are defined as follows. We denote $(\widehat{F}_{T,\lambda ,\alpha ,n}^{(k)})_{k \in \{1, \ldots , M \}}$, $(\widehat{F}_{C,\lambda ,\beta ,n}^{(k)})_{k \in \{1, \ldots , M \}}$ the marginal estimated distribution functions and $F_T$, $F_C$ the true marginal distribution functions. The *nx* and *bias* functions are defined as follows:

$$\begin{aligned} nx_T (t)= & \frac{1}{M} \sum _{k=1}^M (\widehat{F}_{T,\lambda ,\alpha ,n}^{(k)} (t)- F_T (t))^2\\ \text {and} & \\ bias_T (t)= & \frac{1}{M} \sum _{k=1}^M | \widehat{F}_{T,\lambda ,\alpha ,n}^{(k)} (t) - F_T (t) | \quad ; \end{aligned}$$

**Fig. 1:**
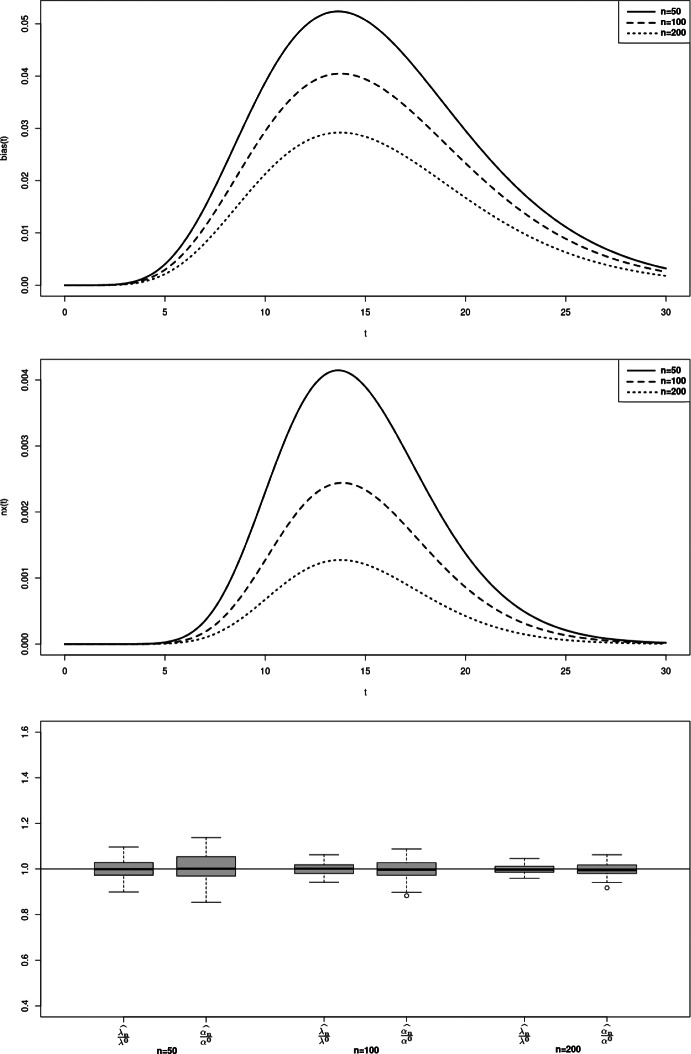
Case a - Bias function $bias_T(t)$ (top), mean squared error function $nx_T(t)$ (middle) and boxplot of M = 100 realisations of the estimator divided by the true value, for n=50, 100, 200

**Fig. 2:**
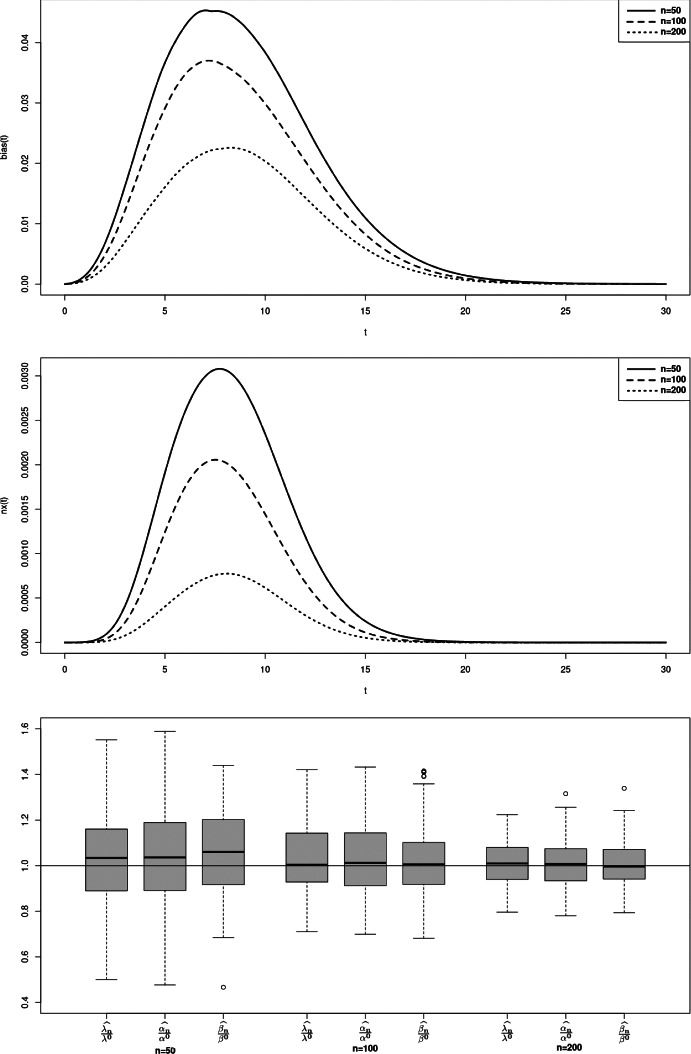
Case b - Bias function $bias_T(t)$ (top), mean squared error function $nx_T(t)$ (middle) and boxplot of M = 100 realisations of the estimator divided by the true value, for n=50, 100, 200

**Fig. 3:**
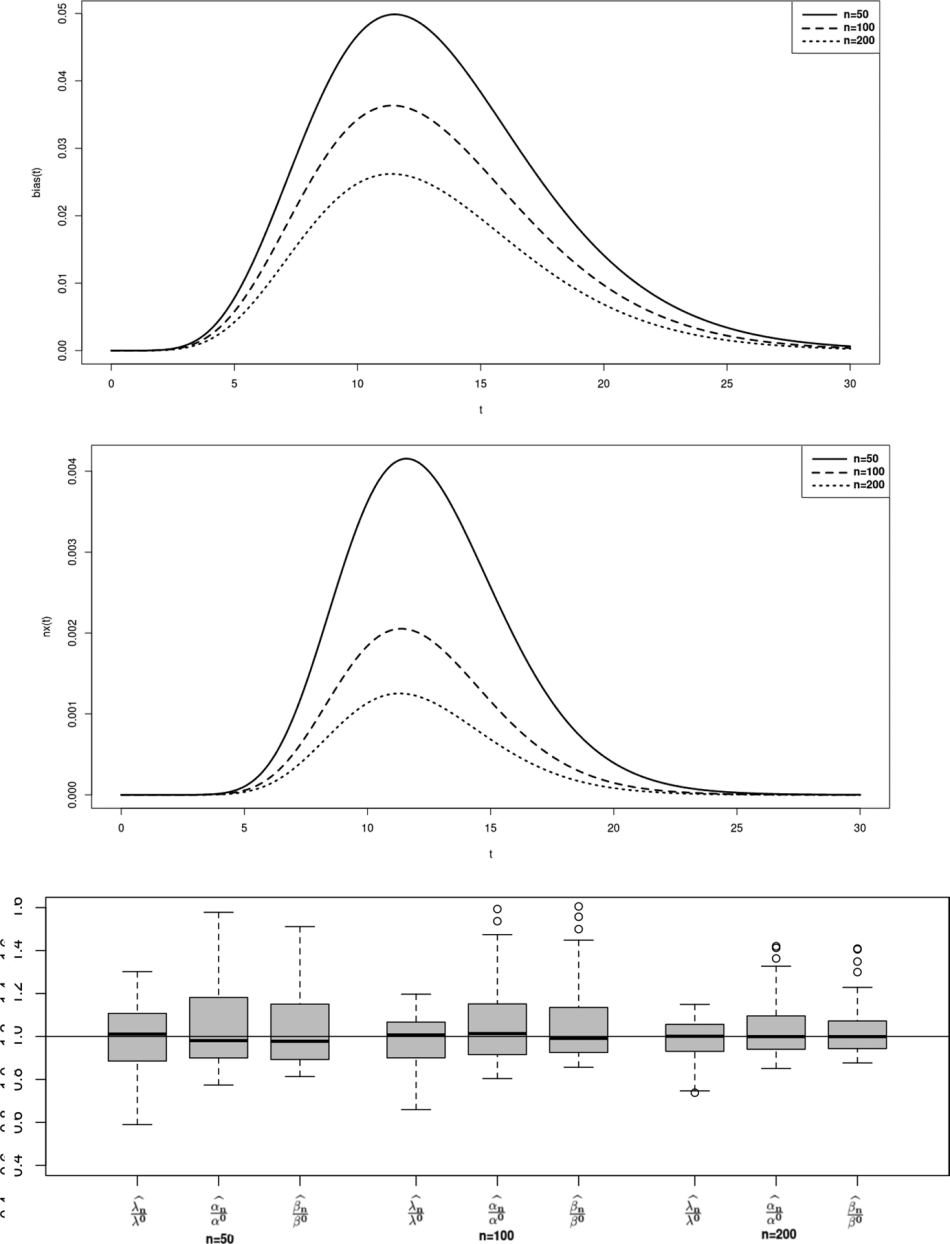
Case c - Bias function $bias_T(t)$ (top), mean squared error function $nx_T(t)$ (middle) and boxplot of M = 100 realisations of the estimator divided by the true value, for n=50, 100, 200

$nx_C$ and $bias_C$ are defined the same way. To illustrate the property of asymptotic normality, we also present rejection rates based on the estimation of the model and empirical matrices of the limiting distribution. For any symmetric positive definite matrix *A*, we denote $\sqrt{A}$ its unique Cholesky decomposition matrix with positive diagonal components. Boxplots from figures 1-3 illustrate the consistency of the estimator. Contrarily to the case a., the cases b. and c. require to estimate three parameters instead of two, inducing a larger variance of the estimation. The results in the Tables 1 and 2 then return the proportion of random variables $(\Vert \sqrt{nA_n}(\theta _n^k - \theta )\Vert _2^2)_{k=1,\ldots ,M}$ that do not exceed quantiles of the $\chi ^2$ distribution with $d_1 + d_2 + 1$ degrees of freedom, where $A_n$ refers to either $B_n(\theta _n^k)$ or the inverse of the empirical covariance matrix $D_{M,n}$.

In those examples, the orders of the partial sum of the density function need to be determined to ensure the consistency while optimizing/minimizing the time of computation. Density function from case a. is already a finite sum, leading to considering the true function for the estimation. For case b. and c., we respectively consider the partial sums of order $N = 25$ and $N=28$ (Figs. 1, 2, 3).

**Table 1:** Case a - Acceptation rates for $90\%$, $95\%$, $99\%$-quantiles of a $\chi _2$ distribution, for $M=100$ realisations of the estimator, considering either $D_{M,n}$ and $B_n(\theta _n)$, for $n=50,100,200,400,800$

Matrix	$n=50$	$n=100$	$n=200$	$n=400$	$n=800$	Quantile ($\%$)
$D_{M,n}$	.91	.92	.91	.89	.92	90
$B_n(\theta _n)$	.92	.94	.91	.92	.89	
$D_{M,n}$	.95	.97	.94	.96	.96	95
$B_n(\theta _n)$	.97	.98	.96	.96	.95	
$D_{M,n}$	.99	1	1	1	.99	99
$B_n(\theta _n)$	.99	1	1	.99	.98	

**Table 2:** Case b - Acceptation rates for $90\%$, $95\%$, $99\%$-quantiles of a $\chi _2$ distribution, for $M=100$ realisations of the estimator, considering either $D_{M,n}$ and $B_n(\theta _n)$, for $n=50,100,200,400,800$

Matrix	$n=50$	$n=100$	$n=200$	$n=400$	$n=800$	Quantile ($\%$)
$D_{M,n}$	.91	.91	.92	.87	.89	90
$B_n(\theta _n)$	.60	.72	.85	.82	.86	
$D_{M,n}$	.94	.93	.95	.94	.94	95
$B_n(\theta _n)$	.70	.82	.86	.90	.94	
$D_{M,n}$	.99	.99	.99	.98	.99	99
$B_n(\theta _n)$	.79	.93	.97	.98	1	

#### Remark 6.1

Computing the model density functions requires to consider approximations through a finite number of terms in the sums, with a great impact on the estimation efficiency. It is indeed worth mentioning that a large number of coefficients would significantly enhance the estimators efficiency, but also drastically increase the computation time of our approach. Extensive simulations in our study has underlined the importance of an appropriate choice of *N*, in order to guarantee a trade-off between the statistical and computing performances. As mentioned in Sect. 3, the choice of *N* can be deduced from Lemma 3.5 selecting the number of coefficients according to $\lambda _m$ and $\tau$. Case b. is for example treated considering $N= 25$. Due to the sample of realisations, we can fix $\lambda _m =2$ and $\tau =20$. The proposed bound from Lemma 3.5 with *j*=18, gives a term dominated by $3\times 10^{-3}$. Similarly, case c. is treated considering $N= 28$, with a remaining term dominated by $3.4\times 10^{-2}$.

### Real data application: case of amanita poisoning

*Scientific context:* Poison control centers regularly respond to mushroom poisonings such as amanita. Toxins present in these mushrooms are a rare but significant cause of acute liver failure. As an effective antidote, N-acetylcysteine is administered when a patient arrives at the hospital, preventing many cases of liver failure. Since it is rare to know the exact amount of amanita ingested, it becomes difficult to anticipate the level of risk. When the liver processes a toxin, it produces an enzyme called alanine aminotransferase (ALAT). Known thresholds for this enzyme are used to measure patient risk. Another commonly used prognostic indicator is Factor V (FV), expressed as a percentage. A healthy liver has a Factor V of $100\%$, while less than $30\%$ requires transplantation. After amanita ingestion, ALAT and FV levels show monotonic behavior until they respectively reach their peak and minimum. However, patient records of ALAT and FV levels are discontinuous and spaced at random times. When one of the measurements reaches critical levels, decisions must be made to operate the patient. Therefore, it is common to have censored times during follow-up.

To describe the evolution of ALAT and FV levels after ingestion, we consider two monotonic random processes. The measurements of these quantities are performed simultaneously. The times at which patients are tested are random, and we can assume that the inter-periods follow independent random variables with a common exponential distribution. These properties lead us to believe that our model is a suitable candidate to describe the dynamics of ALAT and FV.

*Mathematical description:* For each individual, we consider the processes $L_1$ and $1-L_2$ which describe the levels of ALAT and FV respectively. Since everyone has a different range of ALAT values, we assume $L_1$ represents rescaled levels between 0 and 1, so it maximizes near 1. Based on the patient protocol, a recorded time is of interest if it returns the moment a patient reaches his maximum ALAT or when his FV drops below 30 $\%$. Understanding the distribution of these moments therefore provides important information for patient monitoring and care. Because treatment effects typically occur a few days after administration, it is common to have censored data with sometimes equal recording times for ALAT and FV events.

In this study, we collected $N=81$ patient data with a complete record of time to ALAT maximum and FV drop. The latter are denoted $(T_i)_{1\le i\le N}$ and $(C_i)_{1\le i\le N}$, respectively, and are used to construct the censored dataset $\{(Y_i,\Delta _i), i \in \{1,\dot{,}N\}$ based on the **Model I**. The process are formally described by

$$\begin{aligned} L_{t,1} = \sum _{n=1}^{N_t} X_n\quad \text {and}\quad L_{t,2} = \sum _{n=1}^{N_t} Z_n, \qquad \forall t \ge 0. \end{aligned}$$

where we assume that the jump sizes follows exponential distributions with parameters $\alpha$ and $\beta$ respectively. According to the aforementioned protocol, we consider the thresholds $x=0.95$ and $z= 0.65$. It is worth mentioning that in this configuration, the exponential parameters and the thresholds share some proportional stability, in the sense that $\alpha x$ and $\beta z$ are constants. This property follows from the stability of exponential distributions to scalar multiplications and was observed in our analysis.

**Fig. 4:**
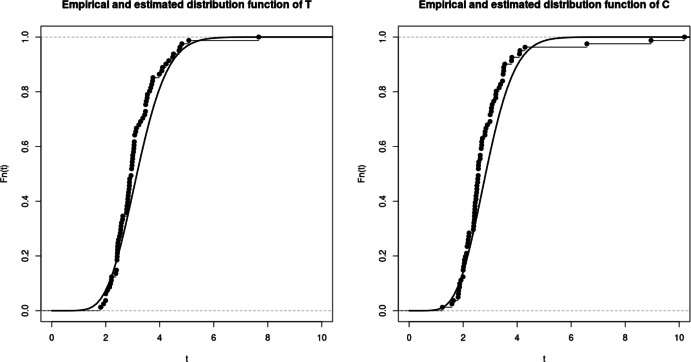
Plot of the estimated (line) and empirical (step function with knots) cumulative distribution functions of T (left) and C (right)

**Fig. 5:**
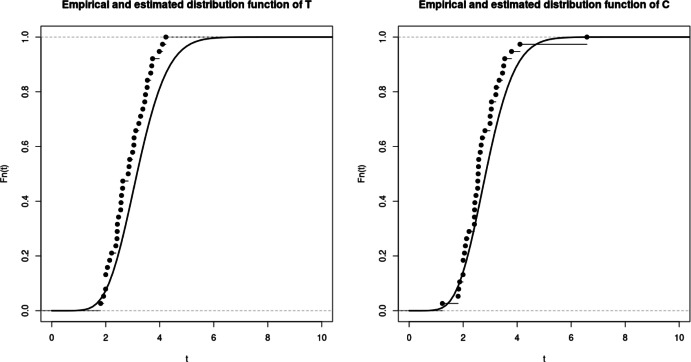
Plot of the estimated (line) and empirical (step function with knots) cumulative distribution functions of T (left) and C (right) for patient younger than 59 years old

**Fig. 6:**
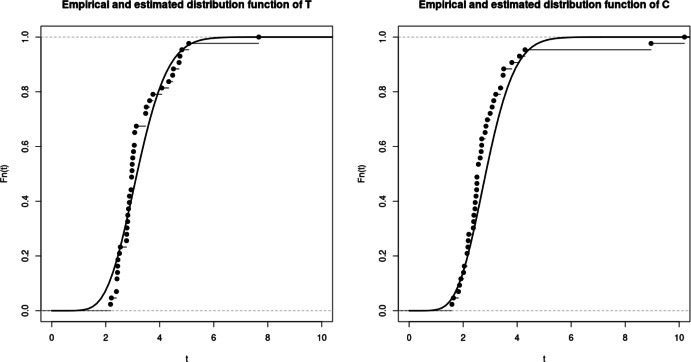
Plot of the estimated (line) and empirical (step function with knots) cumulative distribution functions of T (left) and C (right) for patient older than 60 years old

**Fig. 7:**
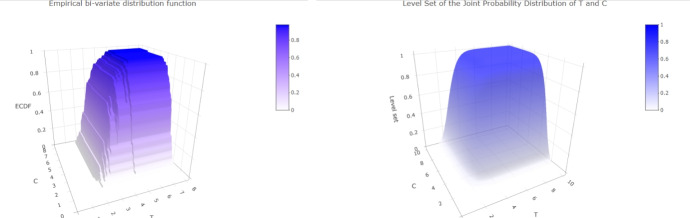
Plot of the empirical (left side) and estimated (right side) joint probability distribution of T and C

Table 4 shows the numerical results of the estimators for different subsamples of the dataset. The abbreviations All, M, F, $A_<$, $A_>$, $CVR_-$ and $CVR_+$ respectively denote all data, male, female, patients below 59 years old, patients above 60 years old, patients with and without history of cerebrovascular risk. The different subsample sizes are presented in Table 3. The probabilities of censoring are estimated throughout the empirical proportions $\mathbb {P}_{\text {Emp}}[ T \le C]$ or based on our model with $\mathbb {P}_{\text {Est}}[ T \le C]$. The curves of the distribution functions obtained from our model are compared with their empirical counterparts in Fig. 4. Similar plots are shown in Figs. 5 and 6, where the population is divided into patients over and under the age of 59. In Fig. 7, we illustrate the dependence structure of the couple (*T*, *C*) with an estimate of the bivariate distribution function based on our model.

**Table 3:** Group sizes of the different subsamples of the dataset

Type	All	M	F	$A_<$	$A_>$	$CVR_-$	$CVR_+$
Group size	81	43	38	38	43	55	26

**Fig. 8:**
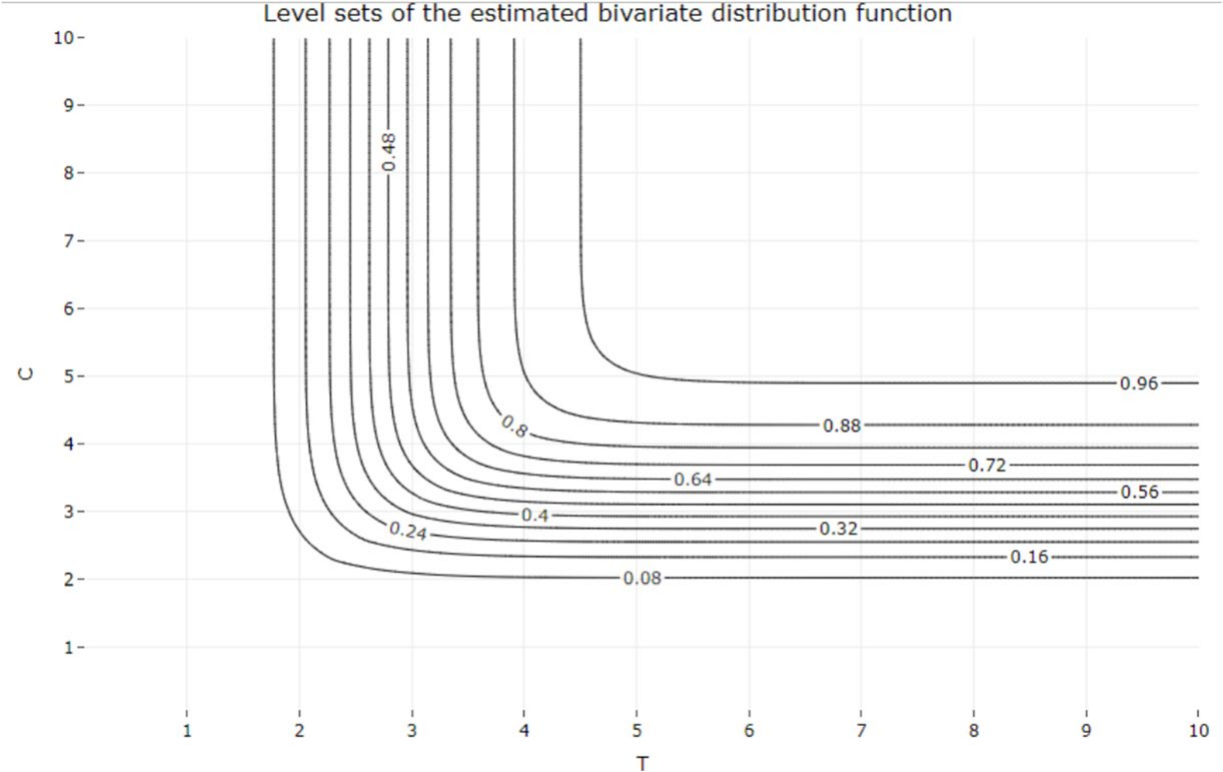
Plot of the level sets of the estimated joint probability distribution of T and C

**Table 4:** Parameter estimation result in accordance with threshold values and population categorization

Threshold (*x*, *z*)	Type	$\widehat{\lambda }_n$	$\widehat{\alpha }_n$	$\widehat{\beta }_n$	$\mathbb {P}_{\text {Emp}}[ T \le C]$	$\mathbb {P}_{\text {Est}}[ T \le C]$
(9.5, 6.5)	All	7.889	2.5673	3.3602	0.3950617	0.381685
(4.75, 3.9)	All	7.889	5.1346	5.6003	0.3950617	0.381685
(0.95, 0.65)	All	7.889	25.673	33.602	0.3950617	0.381685
(0.95, 0.65)	M	7.889	25.673	33.602	0.3684211	0.381685
(0.95, 0.65)	F	7.889	25.673	33.602	0.4186047	0.381685
(0.95, 0.65)	$A_<$	7.889	25.673	33.602	0.5	0.381685
(0.95, 0.65)	$A_>$	7.889	25.673	33.602	0.3023256	0.381685
(0.95, 0.65)	$CVR_-$	7.889	25.673	33.602	0.4	0.381685
(0.95, 0.65)	$CVR_+$	7.889	25.673	33.602	0.3846154	0.381685

The first three lines of the table underline the proportional stability of the exponential law mentioned above. Furthermore, it is worth noting that the estimated parameter $\widehat{\lambda }_n$ remains unchanged when the thresholds are changed, since it only affects the distribution of the jump sizes of both processes. Such a phenomenon is coherent since the expected number of observations needed to observe ALAT or FV peaking remains unchanged throughout the records. The last four rows also illustrate the accuracy of the estimator whether the group is categorized or not, especially with subcategories containing only 26 patients. Although our model do not take into consideration the value of the peaks for each patient, these categorizations show that parameters such as sex, age, history of cerebrovascular risk have no impact on the moment of reaching the peak of ALAT and FV. In particular, our estimator still provides a good estimate even with reduced dataset sizes. This is also observed with comparison between the empirical and modeled curves in Figs. 4, 5 and 6 since we observe few discrepancies between the results. The curves from our model seem to correctly interpret all event times, even though it is based on censored data, and particularly for the largest observations. Lastly, the dependence structure of the couple (*T*, *C*) is presented in the Figs. 7 and 8 with a 3D plot and the plot of levels respectively. The property of non-absolute continuity of the pair can be observed in the left plot with abrupt changes in the curve trajectories in the set $\{(u,v)\in \mathbb {R}^2_+,\, u=v \}$. This was expected from the theoretical model construction and analysis, and reflects the possibility of equal recording times among the data.

Despite the limited number of patients in the dataset, these findings highlight the effectiveness of our model and suggest independence between the peak distributions and patient categories. This has been particularly helpful for the medical team, sparking discussions about developing a model extension in which events are also indexed by compound Poisson processes.

## Supplementary Information

Below is the link to the electronic supplementary material.

Supplementary file 1 (pdf 382 KB)
