# Supplementary material for: A dependent and censored first hitting-time model with compound Poisson processes
Source: Lifetime Data Anal. 2026 Apr 27;32(2):29. doi: 10.1007/s10985-026-09705-1 (PMC13121319; doi:10.1007/s10985-026-09705-1)
Supplement: Supplementary file 1 — (pdf 382 KB) [file 10985_2026_9705_MOESM1_ESM.pdf]

Supplementary material for "A dependent and censored first hitting-time model with compound Poisson processes"

This document regroups the proofs of the main article.

Proof of Lemma 3.1. Firstly, let us compute the distribution function of the couple of hitting time (T, C). We have for $u \geq v$,

$$\mathbb{P}[T \leq u, C \leq v]$$
$$= \sum_{n=1}^{+\infty} \sum_{j=1}^{n} \mathbb{P}[X_1 + \ldots + X_n \geq x]\mathbb{P}[Z_1 + \ldots + Z_j \geq z] e^{-\lambda u} \frac{(\lambda u)^n}{n!} \binom{n}{j} \left(\frac{v}{u}\right)^j \left(1 - \frac{v}{u}\right)^{n-j},$$

and for $v > u$,

$$\mathbb{P}[T \leq u, C \leq v]$$
$$= \sum_{j=1}^{+\infty} \sum_{n=1}^{j} \mathbb{P}[X_1 + \ldots + X_n \geq x]\mathbb{P}[Z_1 + \ldots + Z_j \geq z] e^{-\lambda v} \frac{(\lambda v)^j}{j!} \binom{j}{n} \left(\frac{u}{v}\right)^n \left(1 - \frac{u}{v}\right)^{j-n}. \quad (10)$$

Since the proof use the same ideas on both of the subsets $\{0 \leq u \leq v\}$ and $\{0 \leq v < u\}$, we only prove it when $u \geq v$.
Since both processes L_1 and L_2 are non-decreasing processes, using the total probability formula and using the fact that those processes are subordinators

we obtain

$$
\begin{aligned}
&\mathbb{P}[T \leq u, C \leq v] \\
=&\mathbb{P}[L_{u,1} \geq x, L_{v,2} \geq z] \\
=&\sum_{n=1}^{+\infty}\sum_{j=1}^{+\infty} \mathbb{P}[L_{u,1} \geq x; L_{v,2} \geq z; N_v = j; N_u = n] \\
=&\sum_{n=1}^{+\infty}\sum_{j=1}^{n} \mathbb{P}[L_{u,1} \geq x; L_{v,2} \geq z | N_v = j; N_u = n]\mathbb{P}[N_v = j; N_u = n] \\
=&\sum_{n=1}^{+\infty}\sum_{j=1}^{n} \mathbb{P}[X_1 + \ldots + X_n \geq x; Z_1 + \ldots + Z_j \geq z]\mathbb{P}[N_v = j; N_u - N_v = n - j]
\end{aligned}
$$

The change of index in the second sum comes from the fact that since $u \geq v$, necessarily $(N_t)_{t\geq 0}$ has jumped more at the time u than at the time v. By independence of $(X_i)_{i\geq 1}$ and $(Z_i)_{i\geq 1}$ and by the definition of a Poisson process, we obtain

$$
\begin{aligned}
=&\sum_{n=1}^{+\infty}\sum_{j=1}^{n} \mathbb{P}[X_1 + \ldots + X_n \geq x]\mathbb{P}[Z_1 + \ldots + Z_j \geq z] e^{-\lambda v}\frac{(\lambda v)^j}{j!} e^{-\lambda(u-v)}\frac{(\lambda(u-v))^{n-j}}{(n-j)!} \\
=&\sum_{n=1}^{+\infty}\sum_{j=1}^{n} \mathbb{P}[X_1 + \ldots + X_n \geq x]\mathbb{P}[Z_1 + \ldots + Z_j \geq z] e^{-\lambda u}\frac{(\lambda u)^n}{n!}\binom{n}{j}\left(\frac{v}{u}\right)^j\left(1 - \frac{v}{u}\right)^{n-j}
\end{aligned}
$$

The law of the couple (T, C) is not absolutely continuous with respect to the Lebesgue measure on $\mathbb{R}^2$. It is the case outside of the diagonal $\{(u,u),\ u \in \mathbb{R}_+\}$. A simple differentiation is enough to obtain the density function. Along the diagonal, another proof needs to be done.

Density function of the couple (T, C) outside of the diagonal.. We want to prove that the density function f_{ac} of the couple (T, C) on the subset $\{u \neq v\}$ is given by (3). In fact, $(u, v) \in (\mathbb{R}_+)^2 \mapsto \mathbb{P}[T \leq u, C \leq v]$ has a similar structure on $\{u < v\}$ and $\{u > v\}$. Indeed the previous formulas for $\mathbb{P}[T \leq u, C \leq v]$ are symmetric on $(X_n)_{n\in\mathbb{N}}$ - $(Z_n)_{n\in\mathbb{N}}$ and u - v. Thus only the proof on the subset $\{u < v\}$ is detailed.

On the subset $\{u < v\}$, the distribution function is given by (10) and can be rewritten as

$$\sum_{n=1}^{+\infty}\sum_{j=1}^{n}[1-c_{j,X}][1-c_{n,Z}]e^{-\lambda v}\frac{(\lambda(v-u))^{n-j}}{(n-j)!}\frac{(\lambda u)^j}{j!}.$$

To facilitate the calculus, we can separate this formula in two terms: a first one where $j < n$ and a second one where $j = n$. Then, we get

$$\begin{aligned}\mathbb{P}[T \leq u, C \leq v] =& \sum_{n=2}^{+\infty}\sum_{j=1}^{n-1}[1-c_{j,X}][1-c_{n,Z}]e^{-\lambda v}\frac{(\lambda(v-u))^{n-j}}{(n-j)!}\frac{(\lambda u)^j}{j!}\\ &+\sum_{n=1}^{+\infty}[1-c_{n,X}][1-c_{n,Z}]e^{-\lambda v}\frac{(\lambda u)^n}{n!}.\end{aligned}$$

Denote $A(u, v)$ the first term and $B(u, v)$ the second term. We obtain

$$\begin{aligned}&\partial_v A(u,v)\\ &= \sum_{n=2}^{+\infty}\sum_{j=1}^{n-1}[1-c_{j,X}][1-c_{n,Z}]\lambda e^{-\lambda v}\left[\frac{(\lambda(v-u))^{n-j-1}}{(n-j-1)!}-\frac{(\lambda(v-u))^{n-j}}{(n-j)!}\right]\frac{(\lambda u)^j}{j!}.\end{aligned}$$

By distributing in to distinct sums and applying the index change $n' = n-1$ to the first sum we get

$$\begin{aligned}\partial_v A(u,v) \;=&\; \sum_{n'=1}^{+\infty}\sum_{j=1}^{n'}[1-c_{j,X}][1-c_{n'+1,Z}]\lambda e^{-\lambda v}\frac{(\lambda(v-u))^{n'-j}}{(n'-j)!}\frac{(\lambda u)^j}{j!}\\ &-\sum_{n=2}^{+\infty}\sum_{j=1}^{n-1}[1-c_{j,X}][1-c_{n,Z}]\lambda e^{-\lambda v}\frac{(\lambda(v-u))^{n-j}}{(n-j)!}\frac{(\lambda u)^j}{j!}.\end{aligned}$$

Moreover

$$\partial_v B(u,v) \;=\; -\sum_{n=1}^{+\infty}[1-c_{n,X}][1-c_{n,Z}]\lambda e^{-\lambda v}\frac{(\lambda u)^n}{n!}.$$

Summing the second member of $\partial_v A(u; v)$ and $\partial_v B(u; v)$, we obtain

$$= -\sum_{n=1}^{+\infty}\sum_{j=1}^{n}[1-c_{j,X}][1-c_{n,Z}]\lambda e^{-\lambda v}\frac{(\lambda(v-u))^{n-j}}{(n-j)!}\frac{(\lambda u)^j}{j!}.$$

Finally

$$\begin{aligned}\partial_v \mathbb{P}[T \leq u, C \leq v] &= \partial_v A(u,v) + \partial_v B(u,v) \\ &= \sum_{n=1}^{+\infty}\sum_{j=1}^{n}[1-c_{j,X}][c_{n,Z}-c_{n+1,Z}]\lambda e^{-\lambda v}\frac{(\lambda(v-u))^{n-j}}{(n-j)!}\frac{(\lambda u)^j}{j!}.\end{aligned}$$

The same way as the differentiation on v, to differentiate on u we can concatenate $\partial_v \mathbb{P}[T \leq u, C \leq v]$ in two parts, a first member where $j = n$ and another one where $j < n$.

$$\begin{aligned}\partial_v \mathbb{P}[T \leq u, C \leq v] =& \sum_{n=1}^{+\infty}[1-c_{n,X}][c_{n,Z}-c_{n+1,Z}]\lambda e^{-\lambda v}\frac{(\lambda u)^n}{n!} \\ &+ \sum_{n=2}^{+\infty}\sum_{j=1}^{n-1}[1-c_{j,X}][c_{n,Z}-c_{n+1,Z}]\lambda e^{-\lambda v}\frac{(\lambda(v-u))^{n-j}}{(n-j)!}\frac{(\lambda u)^j}{j!}\end{aligned}$$

The same method (differentiation, separation in two members, change of index) allows us to prove the wanted result for $u < v$.

$$f_{ac}(u,v) = \sum_{n=1}^{+\infty}\sum_{j=1}^{n}\left[c_{j-1,X}-c_{j,X}\right]\left[c_{n,Z}-c_{n+1,Z}\right]\lambda^2 e^{-\lambda v}\frac{(\lambda(v-u))^{n-j}}{(n-j)!}\frac{(\lambda u)^{j-1}}{(j-1)!}.$$

We are not able to use the same method (derivation of the distribution function) to determine the density along the diagonal. Indeed the law of the couple involves a part that is absolutely continuous with respect to the Lebesgue measure and a part that is singular.

Determination of the density function along the diagonal.. The first step of the proof consists of calculating the following distribution function: $u \mapsto \mathbb{P}[T \leq u, T = C]$. We have

$$\begin{aligned}\mathbb{P}[T \leq u, T = C] &= \sum_{k=1}^{+\infty}\mathbb{P}[T \leq u, T = C, N_u = k] \\ &= \sum_{k=1}^{+\infty}\mathbb{P}[T \leq u, T = C | N_u = k]\mathbb{P}[N_u = k]\end{aligned}$$

Then we define the event $\{T \leq u, T = C\}$ using the sequences X_n and Z_n:

$$\begin{aligned}
\mathbb{P}[T \leq u, T = C] = & \sum_{k=1}^{+\infty} \mathbb{P}[\exists n \in \{1, \ldots, k\}, X_0 + \ldots + X_{n-1} < x \leq X_0 + \ldots + X_n; \\
& \qquad Z_0 + \ldots + Z_{n-1} < z \leq Z_0 + \ldots + Z_n]\mathbb{P}[N_u = k] \\
= & \sum_{k=1}^{+\infty} \sum_{n=1}^{k} [c_{n-1,X} - c_{n,X}][c_{n-1,Z} - c_{n,Z}]\mathbb{P}[N_u = k] \\
= & \sum_{n=1}^{+\infty} \sum_{k=n}^{+\infty} [c_{n-1,X} - c_{n,X}][c_{n-1,Z} - c_{n,Z}]\mathbb{P}[N_u = k] \\
= & \sum_{n=0}^{+\infty} \sum_{k=n+1}^{+\infty} [c_{n,X} - c_{n+1,X}][c_{n,Z} - c_{n+1,Z}]\mathbb{P}[N_u = k] \\
= & \sum_{n=0}^{+\infty} [c_{n,X} - c_{n+1,X}][c_{n,Z} - c_{n+1,Z}] e^{-\lambda u} \sum_{k=n+1}^{+\infty} \frac{(\lambda u)^k}{k!}. \qquad (11)
\end{aligned}$$

Differentiating this function leads to the announced result (Equation (4)) and achieves the proof of Lemma 3.1. □

In order to prove Lemma 3.2, we need the following lemma that describes the behavior of the probability terms that appear as coefficient in the density function.

Lemma 1. Let $(X_n)_{n\in\mathbb{N}^*}$ be a sequence of real non-negative i.i.d. random variables. Let $X_0 := 0$ $\mathbb{P}$-as be independent of the sequence $(X_n)_{n\in\mathbb{N}^*}$. Let $x > 0$. Assume that for some $n \in \mathbb{N}$,

$$\mathbb{P}[X_0 + \ldots + X_n < x] = \mathbb{P}[X_0 + \ldots + X_{n+1} < x].$$

Then $\mathbb{P}[X_0 + \ldots + X_n < x] = 0$ or $\mathbb{P}[X_0 + \ldots + X_n < x] = 1$.

Proof of Lemma 1. Define $S_n = \sum_{k=0}^{n} X_k, n \in \mathbb{N}$. Assume that for some $n \in \mathbb{N}$

$$\mathbb{P}[S_n < x] = \mathbb{P}[S_{n+1} < x].$$

To prove this result, we are reasoning reductio ad absurdum. Assume that $0 < \mathbb{P}[S_n < x] < 1$. We first observe that the support of X_1 is a subset of $[0, x[$. If it is not the case, then

$$\mathbb{P}[S_{n+1} < x] \leq \mathbb{P}[X_0 + \ldots + X_n < x]\mathbb{P}[X_1 < x] < \mathbb{P}[S_n < x].$$

Let us define $M_{\max} = \sup\{t \in Supp\{X_1\}\}$. In our setting, $M_{\max} > 0$ (X_1 is not reduced to zero) which proves that $M_{\max} \le x$.
We also claim that $Supp\{S_n\} \cap [0, x[\subset [0, x - M_{\max}[$. If it is not the case, i.e.

$$\mathbb{P}[x - M_{\max} \le S_n < x] > 0, \tag{12}$$

then

$$\begin{aligned}\mathbb{P}[S_{n+1} < x] &= \mathbb{P}[S_{n+1} < x; S_n < x - M_{\max}] + \mathbb{P}[S_{n+1} < x; S_n \ge x - M_{\max}] \\ &= \mathbb{P}[S_n < x - M_{\max}] + \mathbb{P}[S_{n+1} < x; S_n \ge x - M_{\max}]. \end{aligned} \tag{13}$$

The study of the second term must be treated by distinguishing the two following cases.

- Assume that $\mathbb{P}[x - M_{\max} \le S_n < x] > \mathbb{P}[S_n = x - M_{\max}]$. Then there necessarily exists $t \in]x - M_{\max}, x[$ such that $\mathbb{P}[x - M_{\max} \le S_n < x] > \mathbb{P}[t \le S_n < x]$ and we deduce that

  $$\begin{aligned}&\mathbb{P}[S_{n+1} < x; S_n \ge x - M_{\max}] \\ &= \mathbb{P}[S_{n+1} < x; S_n \ge t] + \mathbb{P}[S_{n+1} < x; x - M_{\max} \le S_n < t] \\ &\le \mathbb{P}[X_{n+1} < x - t; t \le S_n < x] + \mathbb{P}[S_{n+1} < x; x - M_{\max} \le S_n < t] \\ &= \mathbb{P}[X_{n+1} < x - t]\mathbb{P}[t \le S_n < x] + \mathbb{P}[S_{n+1} < x; x - M_{\max} \le S_n < t] \\ &< \mathbb{P}[t \le S_n < x] + \mathbb{P}[S_{n+1} < x; x - M_{\max} \le S_n < t] \\ &< \mathbb{P}[x - M_{\max} \le S_n < x].\end{aligned}$$

  We used that $\mathbb{P}[X_{n+1} < x - t] < 1$, since $x - t < M_{\max}$. With (13), we obtatin that $\mathbb{P}[S_{n+1} < x] < \mathbb{P}[S_n < x]$, which contradicts the assumption of this lemma.

  As a consequence, we obtain that (12) becomes

  $$\mathbb{P}[x - M_{\max} \le S_n < x] = \mathbb{P}[x - M_{\max} = S_n] > 0.$$

- Assume that $\mathbb{P}[x - M_{\max} \le S_n < x] = \mathbb{P}[S_n = x - M_{\max}] > 0$. Define $\mathrm{Atom}(X_1) = \{t \ge 0, \mathbb{P}[X_1 = t] > 0\}$ the set of atoms of X_1. Remark that $\mathbb{P}[S_n = x - M_{\max}] \ne 0$ implies that $\mathrm{Atom}(X_1)$ is not empty. Moreover we obtain the following equality

  $$\begin{aligned}\mathbb{P}[S_{n+1} < x; S_n \ge x - M_{\max}] &= \mathbb{P}[X_{n+1} < M_{\max}; S_n = x - M_{\max}] \\ &= \mathbb{P}[X_{n+1} < M_{\max}]\mathbb{P}[S_n = x - M_{\max}].\end{aligned}$$

If $M_{\max}$ is an atom of X_1, then $\mathbb{P}[X_{n+1} < M_{\max}] < 1$ and thus with (13), $\mathbb{P}[S_{n+1} < x] < \mathbb{P}[S_n < x]$, which is again absurd. Therefore $M_{\max}$ is not an atom of X_1 and for any atom a, $\mathbb{P}(X_1 = a) < \mathbb{P}(a \leq X_1 < M_{\max})$. Define the set $A_n = \{(x_1, \ldots, x_n) \in \text{Atom}(X_1)^n; x_1 + \ldots + x_n = x - M_{\max}\}$. We get

$$\begin{aligned}\mathbb{P}[S_n = x - M_{\max}] &= \sum_{(x_1,\ldots,x_n)\in A_n} \mathbb{P}[X_1 = x_1; \ldots; X_n = x_n] \\ &< \sum_{(x_1,\ldots,x_n)\in A_n} \mathbb{P}[X_1 = x_1; \ldots; x_n \leq X_n < M_{\max}] \\ &\leq \mathbb{P}[x - M_{\max} \leq S_n < x].\end{aligned}$$

This contradicts the fact that $\mathbb{P}[x - M_{\max} \leq S_n < x] = \mathbb{P}[S_n = x - M_{\max}]$.

We conclude that $Supp\{S_n\} \cap [0, x[\subset [0, x - M_{\max}[$, i.e. $\mathbb{P}[S_n < x] = \mathbb{P}[S_n < x - M_{\max}]$. Since $0 < \mathbb{P}[S_n < x]$, we must have $M_{\max} < x$.

Our assumption that $\mathbb{P}[S_n < x] < 1$ implies that $nM_{\max} \geq x$. But we have

$$\begin{aligned}\mathbb{P}[S_n < x - M_{\max}] &\leq \mathbb{P}[S_{n-1} < x - M_{\max}] \\ &= \mathbb{P}[S_{n-1} < x - M_{\max}]\mathbb{P}[X_n \leq M_{\max}] \\ &= \mathbb{P}[S_{n-1} < x - M_{\max}; X_n \leq M_{\max}] \\ &\leq \mathbb{P}[S_n < x] = \mathbb{P}[S_n < x - M_{\max}].\end{aligned}$$

Hence

$$0 < \mathbb{P}[S_n < x - M_{\max}] = \mathbb{P}[S_{n-1} < x - M_{\max}] < 1.$$

Using the same ideas of the beginning of the proof, we obtain that $Supp\{S_{n-1}\} \cap [0, x - M_{\max}[\subset [0, x - 2M_{\max}[$ and $\mathbb{P}[S_{n-1} < x - M_{\max}] = \mathbb{P}[S_{n-1} < x - 2M_{\max}]$. Recursively we deduce that $\mathbb{P}[S_n < x] = \mathbb{P}[X_1 < x - nM_{\max}] = 0$, or that $x \leq nM_{\max} < x$, which is absurd. The conclusion of the lemma follows.

$$\mathbb{P}[S_n < x] = \mathbb{P}[S_{n+1} < x] = 0 \quad \text{or} \quad 1.$$

□

Proof of Lemma 3.2. Formula (5)

$$\mathbb{P}\left[T = C\right] = \sum_{n\in\mathbb{N}} [c_{n,X} - c_{n+1,X}][c_{n,Z} - c_{n+1,Z}]$$

is obtained by integrating the density function (4) in Lemma 3.1 along the diagonal $\{(u, v), u, v \geq 0, u = v\}$ or computing the limit at infinity in (11) (note that the function $u \mapsto \sum_{k=n+1}^{+\infty} e^{-\lambda u} \frac{(\lambda u)^k}{k!}$ is the cumulative distribution function of the Erlang law with parameters $(\lambda, n + 1)$).

Assume that $T < C$ almost surely. The case $T > C$ almost surely is treated the same way. We naturally have $\mathbb{P}\left[T = C\right] = 0$. Assume that $\mathbb{P}\left[T = C\right] = 0$. Since all the terms of the series

$$\sum_{n\in\mathbb{N}} [c_{n,X} - c_{n+1,X}][c_{n,Z} - c_{n+1,Z}]$$

are non-negative, we necessarily have for any $n \in \mathbb{N}$,

$$c_{n,X} = c_{n+1,X} \qquad \text{or} \qquad c_{n,Z} = c_{n+1,Z}$$

This situation can be separated in three different cases.

- *Case 1.* If for all $n \in \mathbb{N}$, $\mathbb{P}[X_0 + \ldots + X_n < x] = \mathbb{P}[X_0 + \ldots + X_{n+1} < x]$, then $\mathbb{P}[X_0 + \ldots + X_n < x] = \mathbb{P}[X_0 < x] = 1, \forall n \in \mathbb{N}$ and since $X_1 \geq 0$ a.s. we must have $X_1 = 0$ a.s. which is not possible by assumption made at the beginning of Section 2. The same happens if for all $n \in \mathbb{N}$, $\mathbb{P}[Z_0 + \ldots + Z_n < z] = \mathbb{P}[Z_0 + \ldots + Z_{n+1} < z]$.

- *Case 2.* Suppose that there exists $n_1 < n_2$ with $n_1, n_2 \in \mathbb{N}$ such that $c_{n_1,X} \neq c_{n_1+1,X}$ and $c_{n_2} = c_{n_2+1,X}$. From Lemma 1 we deduce that $c_{n_2,X} = 0$ or $c_{n_2,X} = 1$. But in the second case, we would have for any $n \leq n_2$, $c_{n,X} = 1$ and in particular $c_{n_1,X} = c_{n_1+1,X}$. Therefore $c_{n_2,X} = 0$ and thus for any $n \geq n_2$, $c_{n,X} = 0$ and $X_1 \geq \frac{x}{n_2}$ a.s.

  Notice that since $c_{n_1,X} \neq c_{n_1+1,X}$, necessarily $c_{n_1+1} < 1$ and thus for any $n > n_1$, $c_{n,X} < 1$.

  Now we define the set $J = \{n \in \mathbb{N}, c_{n,X} \neq c_{n+1,X}\}$. This set is non empty ($n_1 \in J$) and bounded (by n_2), hence J admits a maximum $N_{\max}$ and a minimum $N_{\min}$. Again from Lemma 1, we deduce that $J = \{N_{\min}, \ldots, N_{\max}\}$. Remark that the hitting time T occurs almost

surely at or after the $N_{\min}$-th jump of the Poisson process and almost surely at or before the $N_{\max}+1$-th jump of the Poisson process.

In this configuration, for any $n \in J$, $c_{n,Z} = c_{n+1,Z}$. From Lemma 1 we deduce that $c_{N_{\min},Z} = 0$ or 1. In the first case, $Z_1 \geq \frac{z}{N_{\min}}$ and thus the hitting time C occurs almost surely strictly before the $N_{\min}$-th jump of the Poisson process, that is $C < T$ almost surely. If $c_{N_{\min},Z} = 1$, then $c_{N_{\max},Z} = 1$ and a.s. C occurs after the $N_{\max}+1$-th jump of the Poisson process, that $C \geq T$ almost surely.

- *Case 3.* If for any $N \in \mathbb{N}$, there exists $n \geq N$, $c_{n,X} \neq c_{n+1,X}$, using the same type of arguments as in Case 2, we obtain that $C < T$ almost surely.

Lemma 3.2 is proved. □

Proof of Lemma 3.4. The proof for Model II is derived directly from the proof for Model I, so only the latter will be considered in the forthcoming analysis. The first step to determine the density function of the censoring couple relies on the cumulative distribution function $F_{(Y,\Delta)}$. We have

$$F_{(Y,\Delta)}(t,0) = \mathbb{P}(C \leq t, C < T) = \int_{\mathbb{R}^2} \mathbb{1}_{\{0 \leq v \leq t, v < u\}} f_{ac}(u,v)\,du\,dv.$$

Thus

$$\begin{aligned}
&\mathbb{P}[C \leq t, T > C] \\
&= \int_0^t \left(\int_v^\infty \sum_{n=1}^{+\infty} \sum_{j=1}^{n} [c_{n,X} - c_{n+1,X}][c_{j-1,Z} - c_{j,Z}] \lambda^2 e^{-\lambda u} \frac{(\lambda(u-v))^{n-j}}{(n-j)!} \frac{(\lambda v)^{j-1}}{(j-1)!} du \right) dv \\
&= \int_0^t \sum_{n=1}^{+\infty} \sum_{j=1}^{n} [c_{n,X} - c_{n+1,X}][c_{j-1,Z} - c_{j,Z}] \lambda e^{-\lambda v} \frac{(\lambda v)^{j-1}}{(j-1)!} dv \\
&= \sum_{n=1}^{+\infty} \sum_{j=1}^{n} [c_{n,X} - c_{n+1,X}][c_{j-1,Z} - c_{j,Z}] \left[1 - \sum_{k=0}^{j-1} e^{-\lambda t} \frac{(\lambda t)^k}{k!} \right] \\
&= \sum_{\ell=0}^{+\infty} c_{\ell+1,X} [c_{\ell,Z} - c_{\ell+1,Z}] \left[1 - \sum_{k=0}^{\ell} e^{-\lambda t} \frac{(\lambda t)^k}{k!} \right].
\end{aligned}$$

In order to derive $F_{(Y,\Delta)}(t,1)$ for $t \geq 0$, one can see that

$$F_{(Y,\Delta)}(t,1) = \mathbb{P}[T \leq t, T \leq C] = \mathbb{P}[T \leq t] - \mathbb{P}[T \leq t, T > C],$$

where similar arguments as in the proof of Lemma 3.1 gives

$$\mathbb{P}[T \le t] = \sum_{n=1}^{+\infty} [1 - c_{n,X}] e^{-\lambda t} \frac{(\lambda t)^n}{n!}.$$

By use of the density function f_{ac} given by (3), we obtain

$$\begin{aligned}
\mathbb{P}[T \le t, T > C] &= \int_{\mathbb{R}^2} \mathbb{1}_{\{0 \le v < u \le t\}} f_{ac}(u,v) du dv \\
&= \int_0^t \int_0^u \sum_{n=1}^{+\infty} \sum_{j=1}^{n} [c_{n,X} - c_{n+1,X}][c_{j-1,Z} - c_{j,Z}] \lambda^2 e^{-\lambda u} \frac{(\lambda(u-v))^{n-j}}{(n-j)!} \frac{(\lambda v)^{j-1}}{(j-1)!} du dv.
\end{aligned}$$

Computing this integral directly gives

$$F_{(Y,\Delta)}(t,1) = \sum_{n=1}^{+\infty} [1 - c_{n,X}] e^{-\lambda t} \frac{(\lambda t)^n}{n!} - \sum_{n=1}^{+\infty} [c_{n,X} - c_{n+1,X}][1 - c_{n,Z}] \left[1 - \sum_{k=0}^{n} e^{-\lambda t} \frac{(\lambda t)^k}{k!} \right].$$

By differentiating, we obtain the density function and conclude the proof of this lemma. □

Proof of Lemma 3.5. The proof of the uniform bound for Model II uses the same arguments as the proof for Model I. We define $R_{N,(Y,\Delta)}(t,\delta) = f_{(Y,\Delta)}(t,\delta) - f_{N,(Y,\Delta)}(t,\delta)$ the rest of order N of the density function. The cases $\delta = 0$ or 2 are also treated in the same way as the case $\delta = 1$. Thus, we prove the result when we consider the density from Model I, when $\delta = 1$. Let $N \in \mathbb{N}$, $j \in \{0, \ldots, N\}$, $(t, \lambda) \in [0, \tau] \times [0, \lambda_m]$. We have

$$\begin{aligned}
\frac{R_{N,(Y,\Delta)}(t,1)}{f_{N,(Y,\Delta)}(t,1)} &= \frac{\sum_{n=N+1}^{+\infty} [c_{n,X} - c_{n+1,X}] c_{n,Z} \lambda e^{-\lambda t} \frac{(\lambda t)^n}{n!}}{\sum_{n=0}^{N} [c_{n,X} - c_{n+1,X}] c_{n,Z} \lambda e^{-\lambda t} \frac{(\lambda t)^n}{n!}} \\
&\le \frac{j!}{[c_{j,X} - c_{j+1,X}] \, c_{j,Z}} \sum_{n=N+1}^{+\infty} [c_{n,X} - c_{n+1,X}] c_{n,Z} \frac{(\lambda t)^{n-j}}{n!} \\
&\le \frac{j!}{[c_{j,X} - c_{j+1,X}] \, c_{j,Z}} \sum_{n=N+1}^{+\infty} [c_{n,X} - c_{n+1,X}] c_{n,Z} \frac{(\lambda_m \tau)^{n-j}}{n!} \\
&\le \frac{j!}{(\lambda_m \tau)^j} \frac{\sup_{n>N} \{[(c_{n,X} - c_{n+1,X}) c_{n,Z}\}}{[c_{j,X} - c_{j+1,X}] \, c_{j,Z}} \sum_{n=N+1}^{+\infty} \frac{(\lambda_m \tau)^n}{n!}.
\end{aligned}$$

Then we obtain that

$$\log(f_{(Y,\Delta)}(t,\delta)) = \log(f_{N,(Y,\Delta)}(t,\delta)+R_{N,(Y,\Delta)}(t,\delta)) = \log(f_{N,(Y,\Delta)}(t,\delta))+r_N(T,\delta),$$

with

$$r_N(T,\delta) = \log\left(1+\frac{R_{N,(Y,\Delta)}(t,\delta)}{f_{N,(Y,\Delta)}(t,\delta)}\right) \leq \frac{R_{N,(Y,\Delta)}(t,\delta)}{f_{N,(Y,\Delta)}(t,\delta)}.$$

Proof of Lemma 4.3.. The statement of the lemma implicitly assumes that the function $t \mapsto \sum_{n=0}^{\infty} a_n b_n t^n$ is well defined on $\mathbb{R}_+$ and that $a_n > 0$ and $b_n > 0$ for any n.

Now suppose that $\lim_{n\longrightarrow+\infty} \frac{a_{n+1}}{a_n} = a$. Then for all $\varepsilon > 0$, there exists $N \in \mathbb{N}$ such that $\forall n \geq N$, $\left|\frac{a_{n+1}}{a_n} - a\right| \leq \frac{\varepsilon}{2}$. Then for all $t > 0$,

$$\begin{aligned}
&|g(t)-a| \\
=&\left|\frac{\sum_{n=0}^{+\infty} a_{n+1}b_nt^n}{\sum_{n=0}^{+\infty} a_nb_nt^n} - a\frac{\sum_{n=0}^{+\infty} a_nb_nt^n}{\sum_{n=0}^{+\infty} a_nb_nt^n}\right| \\
=&\left|\frac{\sum_{n=0}^{N-1} a_{n+1}b_nt^n}{\sum_{n=0}^{+\infty} a_nb_nt^n} + \frac{\sum_{n=N}^{+\infty} a_{n+1}b_nt^n}{\sum_{n=0}^{+\infty} a_nb_nt^n} - a\frac{\sum_{n=0}^{N-1} a_nb_nt^n}{\sum_{n=0}^{+\infty} a_nb_nt^n} - a\frac{\sum_{n=N}^{+\infty} a_nb_nt^n}{\sum_{n=0}^{+\infty} a_nb_nt^n}\right| \\
\leq&\left|\frac{\sum_{n=0}^{N-1} a_{n+1}b_nt^n}{\sum_{n=0}^{+\infty} a_nb_nt^n} - a\frac{\sum_{n=0}^{N-1} a_nb_nt^n}{\sum_{n=0}^{+\infty} a_nb_nt^n}\right| + \left|\frac{\sum_{n=N}^{+\infty} a_{n+1}b_nt^n}{\sum_{n=0}^{+\infty} a_nb_nt^n} - a\frac{\sum_{n=N}^{+\infty} a_nb_nt^n}{\sum_{n=0}^{+\infty} a_nb_nt^n}\right| \\
=&\left|\frac{\sum_{n=0}^{N-1}(a_{n+1}-aa_n)b_nt^n}{\sum_{n=0}^{+\infty} a_nb_nt^n}\right| + \left|\frac{\sum_{n=N}^{+\infty}\left(\frac{a_{n+1}}{a_n}-a\right)a_nb_nt^n}{\sum_{n=0}^{+\infty} a_nb_nt^n}\right| \\
\leq&\frac{\sum_{n=0}^{N-1}|a_{n+1}-aa_n|b_nt^n}{\sum_{n=0}^{N} a_nb_nt^n} + \frac{\varepsilon}{2}\frac{\sum_{n=N}^{+\infty} a_nb_nt^n}{\sum_{n=0}^{+\infty} a_nb_nt^n} \\
\leq&\frac{\varepsilon}{2}+\frac{\varepsilon}{2}.
\end{aligned}$$

The last inequality comes from the fact that the function is the quotient of a polynomial function of degree $N-1$ and of a polynomial function of degree N, so that

$$\lim_{t\longrightarrow+\infty} \frac{\sum_{n=0}^{N-1}|a_{n+1}-aa_n|b_nt^n}{\sum_{n=0}^{N} a_nb_nt^n} = 0.$$

Finally $\lim_{t\longrightarrow+\infty} g(t) = a$. □

Proof of Theorem 4.6. Assume that $(X_n)_{n\in\mathbb{N}^*} \in F_2$. Denote ϕ the density of X_1 and ϕ_{S_n} the density function of $S_n = \sum_{k=1}^n X_k$, $n \in \mathbb{N}^*$. Let $n \in \mathbb{N}$. Firstly we have

$$\frac{\mathbb{P}[S_{n+1} < x]}{\mathbb{P}[S_n < x]} = \int_0^x \phi(t) \frac{\mathbb{P}[S_n < x-t]}{\mathbb{P}[S_n < x]} dt. \tag{14}$$

We can study the quotient $\dfrac{\mathbb{P}[S_n < x-t]}{\mathbb{P}[S_n < x]}$. Consider N_0 be the minimal integer such that ϕ_{S_N} is non-decreasing on $[0, x]$. Let $n \in \mathbb{N}$, such that $n \geq N_0$. Set up the Euclidean division of n by N_0: $n = qN_0 + r$, where $q > 0$ and $0 \leq r < N_0$. Then

$$\begin{aligned}&\mathbb{P}[X_1 + \ldots + X_n < x - t]\\ &= \mathbb{P}[X_1 + \ldots + X_{N_0} + \ldots + X_{N_0(q-1)+1} + \ldots + X_{N_0 q} + X_{N_0 q+1} + \ldots + X_{N_0 q+r} < x - t].\end{aligned}$$

Since the sequence $(X_i)_{i\in\mathbb{N}^*}$ is i.i.d., the variables $(X_1 + \ldots + X_{N_0})$, ... , $(X_{N_0(q-1)+1} + \ldots + X_{N_0 q})$, $X_{N_0 q+1}$, ... , $X_{N_0 q+r}$ are independent. Then

$$\begin{aligned}&\mathbb{P}[X_1 + \ldots + X_{N_0} + \ldots + X_{N_0(q-1)+1} + \ldots + X_{N_0 q} + X_{N_0 q+1} + \ldots + X_{N_0 q+r} < x - t]\\ &\quad= \int_{(\mathbb{R}+)^{q+r}} \phi_{S_{N_0}}(x_1)\ldots\phi_{S_{N_0}}(x_q)\phi(x_{q+1})\ldots\phi(x_{q+r})\mathbb{1}_{\{0\leq x_1+\ldots+x_{q+r}<x-t\}} dx_1\ldots dx_{q+r}\\ &\quad= \int_{(\mathbb{R}+)^{q+r}} \phi_{S_{N_0}}(x_1)\ldots\phi_{S_{N_0}}(x_q)\phi(x_{q+1})\ldots\phi(x_{q+r})\mathbb{1}_{\{0\leq \frac{x}{x-t}x_1+\ldots+\frac{x}{x-t}x_{q+r}<x\}} dx_1\ldots dx_{q+r}.\end{aligned}$$

By the change of variables $y_i = \frac{x}{x-t}x_i, i \in \{0, ..., q\}$ et $y_i = x_i, i \in \{q+1, ..., q+r\}$, we get

$$\begin{aligned}&\int_{(\mathbb{R}+)^{q+r}} \phi_{S_{N_0}}(x_1)\ldots\phi_{S_{N_0}}(x_q)\phi(x_{q+1})\ldots\phi(x_{q+r})\mathbb{1}_{\{0\leq \frac{x}{x-t}x_1+\ldots+\frac{x}{x-t}x_{q+r}<x\}} dx_1\ldots dx_{q+r}\\ &\quad= \left(\frac{x-t}{x}\right)^q \int_{(\mathbb{R}+)^{q+r}} \phi_{S_{N_0}}\left(\frac{x-t}{x}y_1\right)\ldots\phi_{S_{N_0}}\left(\frac{x-t}{x}y_q\right)\phi(y_{q+1})\ldots\phi(y_{q+r})\\ &\qquad\qquad \times \mathbb{1}_{\{0\leq y_1+\ldots+y_q+\frac{x}{x-t}y_{q+1}+\ldots+\frac{x}{x-t}y_{q+r}<x\}} dy_1\ldots dy_{q+r}.\end{aligned}$$

The condition $\{0 \leq y_1 + \ldots + y_q + \frac{x}{x-t}y_{q+1} + \ldots + \frac{x}{x-t}y_{q+r} < x\}$ implies that for any i, $y_i \in [0, x]$. The non-decreasing property of $\phi_{S_{N_0}}$ on $[0, x]$ implies

the following inequality.

$$\left(\frac{x-t}{x}\right)^q \int_{(\mathbb{R}+)^{q+r}} \phi_{S_{N_0}}\left(\frac{x-t}{x}y_1\right)\dots\phi_{S_{N_0}}\left(\frac{x-t}{x}y_q\right)\phi(y_{q+1})\dots\phi(y_{q+r})$$
$$\times \mathbb{1}_{\{0\le y_1+\ldots+y_q+\frac{x}{x-t}y_{q+1}+\ldots+\frac{x}{x-t}y_{q+r}<x\}}dy_1\dots dy_{q+r}$$
$$\le \left(\frac{x-t}{x}\right)^q \int_{(\mathbb{R}+)^{q+r}} \phi_{S_{N_0}}(y_1)\dots\phi_{S_{N_0}}(y_q)\phi(y_{q+1})\dots\phi(y_{q+r})$$
$$\times \mathbb{1}_{\{0\le y_1+\ldots+y_q+\frac{x}{x-t}y_{q+1}+\ldots+\frac{x}{x-t}y_{q+r}<x\}}dy_1\dots dy_{q+r}.$$

Finally, $\{0 \le y_1+\ldots+y_q+\frac{x}{x-t}y_{q+1}+\ldots+\frac{x}{x-t}y_{q+r} < x\} \subset \{0 \le y_1+\ldots+y_{q+r} < x\}$, since $\frac{x}{x-t} > 1$. So,

$$\left(\frac{x-t}{x}\right)^q \int_{(\mathbb{R}+)^{q+r}} \phi_{S_{N_0}}(y_1)\dots\phi_{S_{N_0}}(y_q)\phi(y_{q+1})\dots f(y_{q+r})$$
$$\times \mathbb{1}_{\{0\le y_1+\ldots+y_q+\frac{x}{x-t}y_{q+1}+\ldots+\frac{x}{x-t}y_{q+r}<x\}}dy_1\dots dy_{q+r}$$
$$\le \left(\frac{x-t}{x}\right)^q \int_{(\mathbb{R}+)^{q+r}} \phi_{S_{N_0}}(y_1)\dots\phi_{S_{N_0}}(y_q)\phi(y_{q+1})\dots\phi(y_{q+r})\mathbb{1}_{\{0\le y_1+\ldots+y_{q+r}<x\}}dy_1\dots dy_{q+r}$$
$$\le \left(\frac{x-t}{x}\right)^q \mathbb{P}[X_1+\ldots+X_{Nq+r}<x] = \left(\frac{x-t}{x}\right)^q \mathbb{P}[X_1+\ldots+X_n<x].$$

To conclude, we obtain

$$\frac{\mathbb{P}[X_1+\ldots+X_n<x-t]}{\mathbb{P}[X_1+\ldots+X_n<x]} \le \left(\frac{x-t}{x}\right)^q.$$

But $q = \frac{n-r}{N_0} > \frac{n-N_0}{N_0} \ge 0$, and since $0 \le \frac{x-t}{x} \le 1$, we finally get

$$\frac{\mathbb{P}[X_1+\ldots+X_n<x-t]}{\mathbb{P}[X_1+\ldots+X_n<x]} \le \left(\frac{x-t}{x}\right)^q \le \left(\frac{x-t}{x}\right)^{\frac{n}{N_0}-1}.$$

By applying the dominated convergence theorem inside equation (14), we obtain the desired result.

Assume that $(X_n)_{n\in\mathbb{N}^*} \in F_3$. Let $x > 0$ and $Atom^* = \{t > 0, \mathbb{P}[X_1 = t] > 0\} = \{x_i,\ i \in \mathbb{N}\}$ be the set of positive atoms of X_1. Define for any $t > 0$,

$$x_{\min} = \inf\{x_i \in Atom^*\}, \quad M_{\max,t} = \left\lceil \frac{t}{x_{\min}} \right\rceil, \quad \Xi_t = \{x_i \in Atom^*; x_i < t\}$$
$$\lozenge_t = \left\{(x_{i_1},\dots,x_{i_k}) \in (Atom^*)^k;\ k \le M_{\max,t} \text{ and } \sum_{j=1}^k x_{i_j} < t\right\}.$$

To simplify the formulas, let us re-write for $n \geq M_{\max,t}$, $k \leq M_{\max,t}$ and $(x_{i_1}, \ldots, x_{i_k}) \in \lozenge_t$,

$$\begin{aligned} & B_{n,t,(x_{i_1},\ldots,x_{i_k})} \\ = \ & \{\exists j_1 < \ldots < j_k \leq n; X_{j_1} = x_{i_1}, \ldots, X_{j_k} = x_{i_k}\} \cap \{\forall i \leq n+1; \forall h \leq k; i \neq j_h; X_i = 0\}. \end{aligned}$$

The sets $\{B_{n,t,(x_{i_1},\ldots,x_{i_k})}, (x_{i_1}, \ldots, x_{i_k}) \in \lozenge_t\}$ are defined to constitute a partition of the event $\{S_n < t\}$. Then consider $n \in \mathbb{N}^*$, $n \geq M_{\max,x}$. We obtain

$$\begin{aligned} \mathbb{P}[S_{n+1} < x] =& \mathbb{P}[S_{n+1} < x; X_{n+1} = 0] + \sum_{x_i \in \Xi_x} \mathbb{P}[S_{n+1} < x; X_{n+1} = x_i] \\ =& \mathbb{P}[X_1 = 0]\mathbb{P}[S_n < x] + \sum_{x_i \in \Xi_x} \mathbb{P}[X_1 = x_i]\mathbb{P}[S_n < x - x_i] \\ \leq& \mathbb{P}[X_1 = 0]\mathbb{P}[S_n < x] + \mathbb{P}[S_n < x - x_{\min}] \sum_{x_i \in \Xi_x} \mathbb{P}[X_1 = x_i] \\ \leq& \mathbb{P}[X_1 = 0]\mathbb{P}[S_n < x] + \mathbb{P}[S_n < x - x_{\min}]. \end{aligned} \tag{15}$$

We prove that there exists $C_x > 0$ such that

$$\frac{\mathbb{P}[S_n < x - x_{\min}]}{\mathbb{P}[S_n < x]} \leq \frac{C_x}{n}.$$

Firstly we have

$$\begin{aligned} & \mathbb{P}[S_n < x - x_{\min}] \\ = & \sum_{k=0}^{M_{\max,x-x_{\min}}} \sum_{(x_{i_1},\ldots,x_{i_k}) \in \lozenge_{x-x_{\min}}} \mathbb{P}[S_n < x - x_{\min}; B_{n,x-x_{\min},(x_{i_1},\ldots,x_{i_k})}] \\ = & \sum_{k=0}^{M_{\max,x-x_{\min}}} \sum_{(x_{i_1},\ldots,x_{i_k}) \in \lozenge_{x-x_{\min}}} \mathbb{P}[B_{n,x-x_{\min},(x_{i_1},\ldots,x_{i_k})}] \\ = & \sum_{k=0}^{M_{\max,x-x_{\min}}} \sum_{(x_{i_1},\ldots,x_{i_k}) \in \lozenge_{x-x_{\min}}} \frac{\mathbb{P}[B_{n,x-x_{\min},(x_{i_1},\ldots,x_{i_k})}]}{\mathbb{P}[B_{n,x,(x_{i_1},\ldots,x_{i_k},x_{\min})}]} \mathbb{P}[B_{n,x,(x_{i_1},\ldots,x_{i_k},x_{\min})}]. \end{aligned}$$

Note that

$$\begin{aligned}
&\mathbb{P}[B_{n,x-x_{\min},(x_{i_1},\dots,x_{i_k})}]\\
=&\sum_{1\le j_1<\dots<j_k\le n}\mathbb{P}[X_{j_1}=x_{i_1},\dots,X_{j_k}=x_{i_k};\forall i\le n+1;\forall h\le k;i\ne j_h;X_i=0]\\
=&\sum_{1\le j_1<\dots<j_k\le n}\mathbb{P}[X_1=0]^{n-k}\prod_{j=1}^{k}\mathbb{P}[X_1=x_{i_j}]\\
=&\binom{n}{k}\mathbb{P}[X_1=0]^{n-k}\prod_{j=1}^{k}\mathbb{P}[X_1=x_{i_j}].
\end{aligned}$$

Moreover

$$\begin{aligned}
&\mathbb{P}[B_{n,x,(x_{i_1},\dots,x_{i_k},x_{\min})}]\\
=&\sum_{1\le j_1<\dots<j_{k+1}\le n}\mathbb{P}[X_{j_1}=x_{i_1},\dots,X_{j_k}=x_{i_k},X_{j_{k+1}}=x_{\min};\forall i\le n+1;\forall h\le k;i\ne j_h;X_i=0]\\
=&\sum_{1\le j_1<\dots<j_{k+1}\le n}\mathbb{P}[X_1=0]^{n-k-1}\mathbb{P}[X_1=x_{\min}]\prod_{j=1}^{k}\mathbb{P}[X_1=x_{i_j}]\\
=&\binom{n}{k+1}\mathbb{P}[X_1=0]^{n-k-1}\mathbb{P}[X_1=x_{\min}]\prod_{j=1}^{k}\mathbb{P}[X_1=x_{i_j}].
\end{aligned}$$

Therefore

$$\frac{\mathbb{P}[B_{n,x-x_{\min},(x_{i_1},\dots,x_{i_k})}]}{\mathbb{P}[B_{n,x,(x_{i_1},\dots,x_{i_k},x_{\min})}]}=\frac{k+1}{n-k}\frac{\mathbb{P}[X_1=0]}{\mathbb{P}[X_1=x_{\min}]}\le\frac{M_{\max,x-x_{\min}}+1}{n-M_{\max,x-x_{\min}}}\frac{\mathbb{P}[X_1=0]}{\mathbb{P}[X_1=x_{\min}]}.$$

To conclude we obtain

$$\begin{aligned}
&\mathbb{P}[S_n<x-x_{\min}]\\
\le&\frac{M_{\max,x-x_{\min}}+1}{n-M_{\max,x-x_{\min}}}\frac{\mathbb{P}[X_1=0]}{\mathbb{P}[X_1=x_{\min}]}\sum_{k=0}^{M_{\max,x-x_{\min}}}\sum_{(x_{i_1},\dots,x_{i_k})\in\Diamond_{x-x_{\min}}}\mathbb{P}[B_{n,x,(x_{i_1},\dots,x_{i_k},x_{\min})}]\\
\le&\frac{M_{\max,x-x_{\min}}+1}{n-M_{\max,x-x_{\min}}}\frac{\mathbb{P}[X_1=0]}{\mathbb{P}[X_1=x_{\min}]}\mathbb{P}[S_n<x]\\
\le&\frac{(M_{\max,x-x_{\min}}+1)^2}{n}\frac{\mathbb{P}[X_1=0]}{\mathbb{P}[X_1=x_{\min}]}\mathbb{P}[S_n<x]
\end{aligned}$$

since $n \geq M_{\max,x} = M_{\max,x-x_{\min}}+1$. We can now consider $C_x = M_{\max,x-x_{\min}}(M_{\max,x-x_{\min}}+1)\frac{\mathbb{P}[X_1=0]}{\mathbb{P}[X_1=x_{\min}]}$. Dividing (15) by $\mathbb{P}[S_n < x]$ we finally get the announced result,

$$\left|\frac{\mathbb{P}[S_{n+1} < x]}{\mathbb{P}[S_n < x]} - \mathbb{P}[X_1 = 0]\right| \leq \frac{C_x}{n}.$$

□

Proof of Theorem 4.7. Given $\theta, \theta' \in \Theta$, assume that

$$f_{(Y,\Delta),\theta}(t,\delta) = f_{(Y,\Delta),\theta'}(t,\delta), \qquad \forall t \geq 0, \forall \delta \in \{0;1\}.$$

The remainder of the proof is given as follows: we use the asymptotic behaviour of the hazard function to prove the intensity parameter λ. We then use the previous identification to deduce that I_n is verified. **Step 1.** The first step consists of proving that $\lambda = \lambda'$.
Under **(H1)**, there exists a positive constant $\kappa_1 > 0$ such that X_1 or Z_1 is greater than κ_1 almost surely. A priori $\kappa_1 = \kappa_1(\alpha, \beta)$ depends on the parameter α or β.

Now if we define $N_x(\alpha) = \inf\{n \in \mathbb{N}^* \mid X_1 + \ldots + X_n \geq x, \text{ a.s.}\}$ and $N_z(\beta) = \inf\{n \in \mathbb{N}^* \mid Z_1 + \ldots + Z_n \geq z, \text{ a.s.}\}$, the quantity $N_{\min}(\alpha,\beta) = \min(N_x(\alpha), N_z(\beta))$ is finite, whatever the values of the parameters α and β. Although $\kappa_1(\alpha,\beta)$ and $\kappa_1(\alpha',\beta')$ could different, we will see that necessarily $N_{\min}(\alpha,\beta) = N_{\min}(\alpha',\beta')$. From Lemma 3.4 we can re-write the density function as

$$f_{(Y,\Delta),\theta}(t,1) = \sum_{n=0}^{N_{\min}(\alpha,\beta)-1} \big[c_{n,X}(\alpha) - c_{n+1,X}(\alpha)\big] c_{n,Z}(\beta) \lambda e^{-\lambda t} \frac{(\lambda t)^n}{n!}.$$

Then for all t, $f_{(Y,\Delta),\theta}(t,1) = f_{(Y,\Delta),\theta'}(t,1)$ if and only if

$$\begin{aligned}\sum_{n=0}^{N_{\min}(\alpha,\beta)-1} & [c_{n,X}(\alpha) - c_{n+1,X}(\alpha)] c_{n,Z}(\beta) \lambda e^{-\lambda t} \frac{(\lambda t)^n}{n!} \\ &= \sum_{n=0}^{N_{\min}(\alpha',\beta')-1} [c_{n,X}(\alpha') - c_{n+1,X}(\alpha')] c_{n,Z}(\beta') \lambda' e^{-\lambda' t} \frac{(\lambda' t)^n}{n!},\end{aligned}$$

which implies for all $t \geq 0$,

$$\begin{aligned}
&\frac{\lambda'}{\lambda} e^{(\lambda'-\lambda)t} \\
&= \frac{\sum_{n=0}^{N_{\min}(\alpha,\beta)-1} [c_{n,X}(\alpha) - c_{n+1,X}(\alpha)] c_{n,Z}(\beta) \frac{(\lambda t)^n}{n!}}{\sum_{n=0}^{N_{\min}(\alpha',\beta')-1} [c_{n,X}(\alpha') - c_{n+1,X}(\alpha')] c_{n,Z}(\beta') \frac{(\lambda' t)^n}{n!}} \\
&\underset{t \longrightarrow +\infty}{\sim} \frac{[c_{N_{\min}(\alpha,\beta)-1,X}(\alpha) - c_{N_{\min}(\alpha,\beta),X}(\alpha)] c_{N_{\min}(\alpha,\beta)-1,Z}(\beta) \frac{(\lambda t)^{N_{\min}(\alpha,\beta)-1}}{(N_{\min}(\alpha,\beta)-1)!}}{[c_{N_{\min}(\alpha',\beta')-1,X}(\alpha') - c_{N_{\min}(\alpha',\beta'),X}(\alpha')] c_{N_{\min}(\alpha',\beta')-1,Z}(\beta') \frac{(\lambda' t)^{N_{\min}(\alpha',\beta')-1}}{(N_{\min}(\alpha',\beta')-1)!}} \\
&\underset{t \longrightarrow +\infty}{\sim} C t^{N_{\min}(\alpha,\beta) - N_{\min}(\alpha',\beta')}
\end{aligned}$$

for some constant $C > 0$. This equivalence at infinity is only possible if $\lambda = \lambda'$ and $N_{min}(\alpha, \beta) = N_{\min}(\alpha', \beta')$.

Under Condition **(H2.i)**, from Theorem 4.6, we deduce that for any $(\alpha, \beta) \in \Theta_1 \times \Theta_2$,

$$\lim_{n \longrightarrow +\infty} \frac{\mathbb{P}_\alpha[X_1 + \ldots + X_{n+1} < x] \mathbb{P}_\beta[Z_1 + \ldots + Z_{n+1} < z]}{\mathbb{P}_\alpha[X_1 + \ldots + X_n < x] \mathbb{P}_\beta[Z_1 + \ldots + Z_n < z]} = 0.$$

Since

$$f_{(Y,\Delta),(\lambda,\alpha,\beta)}(t, \delta) = f_{(Y,\Delta),(\lambda',\alpha',\beta')}(t, \delta), \qquad \forall t \geq 0, \forall \delta \in \{0; 1\}$$

we have for all $t \geq 0$, $h_{Y,(\lambda,\alpha,\beta)}(t) = h_{Y,(\lambda',\alpha',\beta')}(t)$. Thus Lemma 4.3 ensures that the hazard function $h_{Y,\theta}$ given by (6) converges to λ as t tends to infinity. We deduce that $\lambda = \lambda'$.

If Condition **(H2.ii)** holds, from Theorem 4.6, we deduce that for any $(\alpha, \beta) \in \Theta_1 \times \Theta_2$,

$$\lim_{n \longrightarrow +\infty} \frac{\mathbb{P}_\alpha[X_1 + \ldots + X_{n+1} < x]}{\mathbb{P}_\alpha[X_1 + \ldots + X_n < x]} = \lim_{n \longrightarrow +\infty} \frac{c_{n+1,X}(\alpha)}{c_{n,X}(\alpha)} = \mathbb{P}_\alpha[X_1 = 0]$$

and

$$\lim_{n \longrightarrow +\infty} \frac{\mathbb{P}_\beta[Z_1 + \ldots + Z_{n+1} < z]}{\mathbb{P}_\beta[Z_1 + \ldots + Z_n < z]} = \lim_{n \longrightarrow +\infty} \frac{c_{n+1,Z}(\beta)}{c_{n,Z}(\beta)} = \mathbb{P}_\beta[Z_1 = 0].$$

Here we consider the censoring indicator from Model II. In order to use Lemma 4.3, one can see that for all $t \geq 0$,

$$\frac{f_{(Y,\Delta),\theta}(t,2)}{f_{(Y,\Delta),\theta}(t,0)} = \frac{\sum_{n=0}^{+\infty}[c_{n,X}(\alpha)-c_{n+1,X}(\alpha)][c_{n,Z}(\beta)-c_{n+1,Z}(\beta)]\lambda e^{-\lambda t}\frac{(\lambda t)^n}{n!}}{\sum_{n=0}^{+\infty}[c_{n,Z}(\beta)-c_{n+1,Z}(\beta)]c_{n+1,X}(\alpha)\lambda e^{-\lambda t}\frac{(\lambda t)^n}{n!}}$$
$$\frac{f_{(Y,\Delta),\theta}(t,2)}{f_{(Y,\Delta),\theta}(t,1)} = \frac{\sum_{n=0}^{+\infty}[c_{n,X}(\alpha)-c_{n+1,X}(\alpha)][c_{n,Z}(\beta)-c_{n+1,Z}(\beta)]\lambda e^{-\lambda t}\frac{(\lambda t)^n}{n!}}{\sum_{n=0}^{+\infty}[c_{n,X}(\alpha)-c_{n+1,X}(\alpha)]c_{n+1,Z}(\beta)\lambda e^{-\lambda t}\frac{(\lambda t)^n}{n!}}.$$

Then applying Lemma 4.3 to the latter series, we obtain that

$$\lim_{t\longrightarrow+\infty}\frac{f_{(Y,\Delta),\theta}(t,2)}{f_{(Y,\Delta),\theta}(t,0)}$$
$$= \lim_{n\longrightarrow+\infty}\frac{[c_{n,X}(\alpha)-c_{n+1,X}(\alpha)][c_{n,Z}(\beta)-c_{n+1,Z}(\beta)]}{[c_{n,Z}(\beta)-c_{n+1,Z}(\beta)]c_{n+1,X}(\alpha)} = \frac{1}{\mathbb{P}_\alpha[X_1=0]}-1$$

and

$$\lim_{t\longrightarrow+\infty}\frac{f_{(Y,\Delta),\theta'}(t,2)}{f_{(Y,\Delta),\theta'}(t,0)}$$
$$= \lim_{n\longrightarrow+\infty}\frac{[c_{n,X}(\alpha')-c_{n+1,X}(\alpha')][c_{n,Z}(\beta')-c_{n+1,Z}(\beta')]}{[c_{n,Z}(\beta')-c_{n+1,Z}(\beta')]c_{n+1,X}(\alpha')} = \frac{1}{\mathbb{P}_{\alpha'}[X_1=0]}-1$$

which proves that $\mathbb{P}_\alpha[X_1=0]=\mathbb{P}_{\alpha'}[X_1=0]$.

Similarly, straightforward algebra based on the limit at infinity of $f_{(Y,\Delta),\theta}(t,2)/f_{(Y,\Delta),\theta}(t,1)$ allows to obtain $\mathbb{P}_\beta[Z_1=0]=\mathbb{P}_{\beta'}[Z_1=0]$. Finally, by use of the limit at infinity of the hazard function of the random variable Y, we obtain that

$$\lambda(1-\mathbb{P}[X_1=0]\mathbb{P}[Z_1=0]) = \lambda'(1-\mathbb{P}_{\alpha'}[X_1=0]\mathbb{P}_{\beta'}[Z_1=0]).$$

Since $\mathbb{P}_\alpha[X_1=0]\mathbb{P}_\beta[Z_1=0]=\mathbb{P}_{\alpha'}[X_1=0]\mathbb{P}_{\beta'}[Z_1=0]<1$, we deduce that $\lambda=\lambda'$ and show I_∞ (resp. $I_{N_{\max}}$) under the assumption **(H2)** (resp. **(H1)**) by evaluating the successive derivatives of the density functions at $t=0$. □

As explained in Section 5, theorems from section 5 are based on convergence results from [29]. Six assumptions are introduced in this paper and can be verified to obtain the existence, its consistency and its asymptotic normality of the QMLE. In order to prove those properties, we define the assumptions **(H3)** (compactness of the set of parameters) and **(H4)** (regularity conditions) detailed here:

Assumption 2 (Condition **(H4)**)**.** There exist two measures ν_1 and ν_2 respectively defined on the measurable space $(\mathbb{R}_+, \mathcal{B}(\mathbb{R}_+))$ such that:

1. For all $\alpha \in \Theta_1$, $\mathbb{P}_\alpha$ admits a Radon-Nikodym measure with respect to ν_1, $f_1(\, . \, , \alpha) = d\mathbb{P}_\alpha / d\nu_1$ such that:
   (a) For all $u \in Dom(f_1) \cap [0, x]$; $\alpha \mapsto f_1(u, \alpha)$ is continuous on Θ_1.
   (b) There exists a ν_1-measurable and integrable function $\overline{f_1}$ on $[0, x]$ such that $\forall \alpha \in \Theta_1$, $f_1(u, \alpha) \leq \overline{f_1}(u)$, for almost every $u \in [0, x]$.
2. For all $\beta \in \Theta_2$, $\mathbb{P}_\beta$ admits a Radon-Nikodym measure with respect to ν_2, $f_2(\, . \, , \beta) = d\mathbb{P}_\beta / d\nu_2$ such that:
   (a) For all $u \in Dom(f_2) \cap [0, z]$; $\beta \mapsto f_2(u, \beta)$ is continuous Θ_2.
   (b) There exists a ν_2-measurable and integrable function $\overline{f_2}$ on $[0, z]$ such that $\forall \beta \in \Theta_2$, $f_2(u, \beta) \leq \overline{f_2}(u)$, for almost every $u \in [0, z]$.

Proof of Theorem 5.1.. In the proof of this theorem, we only discuss the case where we consider the censoring couple (Y, Δ) from Model I. The proof with the strict censoring couple from Model II uses the exact same arguments. To prove this theorem, we verify if A1 and A2 from [29] are verified.

Assumption A1. In our case, the random vector (Y, Δ) takes values in the Euclidean measurable space $(\mathbb{R}^2, \mathcal{B}(\mathbb{R}^2))$. We consider the measure $v = m \otimes d$, where m denotes the Lebesgue measure on $(\mathbb{R}, \mathcal{B}(\mathbb{R}))$ and $d = \delta_0 + \delta_1$, (where δ_a is the Dirac measure in a). It is clear that the random vector (Y, Δ) admits a measurable Radon-Nikodym density function with respect to v, which is $f_{(Y,\Delta)} = f_{(Y,\Delta),\theta}$ given in Lemma 3.4.

Assumption A2. The model is fully determined by the parameter $\lambda \in \mathbb{R}_+^*$, the respective families of distribution functions $\{\mathbb{P}_\alpha, \alpha \in \Theta_1\}$ and $\{\mathbb{P}_\beta, \beta \in \Theta_2\}$, with Θ_1 and Θ_2 respectively subsets of $\mathbb{R}^{d_1}$ and $\mathbb{R}^{d_2}$.

To ensure Assumption A2, it is required to have a compact subset of parameter, which justifies Condition **(H3)**. It guarantees that $\Lambda \times \Theta_1 \times \Theta_2$ is a compact subset of the Euclidean space $\mathbb{R}^{1+d_1+d_2}$. For any $(\lambda, \alpha, \beta) \in \Lambda \times \Theta_1 \times \Theta_2$, we denote by $F_{(Y,\Delta),(\lambda,\alpha,\beta)}$ the distribution function induced by the parameters vector $\theta = (\lambda, \alpha, \beta)$ and by $\theta^0 = (\lambda^0, \alpha^0, \beta^0) \in \Lambda \times \Theta_1 \times \Theta_2$ the true vector of parameters model. For any $(\lambda, \alpha, \beta) \in \Lambda \times \Theta_1 \times \Theta_2$, $F_{(Y,\Delta),(\lambda,\alpha,\beta)}$ admits a measurable Radon-Nikodym function with respect to v, $f_{(Y,\Delta),(\lambda,\alpha,\beta)} = dF_{(Y,\Delta),(\lambda,\alpha,\beta)}/dv$ (Lemma 3.4).

The continuity in λ of the density function $f_{((Y,\Delta),(\lambda,\alpha,\beta))}$ comes naturally for any $(t, \delta) \in \mathbb{R}_+ \times \{0; 1\}$ (it is a uniformly convergent series of function on any compact). To obtain the continuity in α and β, we need to prove that for any $n \in \mathbb{N}$,

$$\alpha \mapsto c_{n,X}(\alpha) = \mathbb{P}_\alpha \left[\sum_{i=0}^{n} X_i < x \right] \quad \text{and} \quad \beta \mapsto c_{n,Z}(\beta) = \mathbb{P}_\beta \left[\sum_{i=0}^{n} Z_i < z \right]$$

are continuous functions. Since the proofs use the same arguments, we will only prove the result for the first function.
If $n = 0$, the functions are constant functions equal to 1, so they are continuous. Let $n \in \mathbb{N}^*$. We can re-write

$$\mathbb{P}_\alpha \left[\sum_{i=0}^{n} X_i < x \right] = \int_{\mathbb{R}_+^n} \mathbb{1}_{\{0 \leq x_1 + \ldots x_n < x\}} \prod_{i=1}^{n} f_1(x_i, \alpha) d\nu_1(x_1) \ldots d\nu_1(x_n).$$

From **(H4)** 2-1.(a), it comes that for any $(x_1, \ldots, x_n) \in \{(y_1, \ldots, y_n) \in Dom(f_1);\ 0 \leq y_1 + \ldots + y_n < x\}$, $\alpha \mapsto \prod_{i=1}^{n} f_1(x_i, \alpha)$ is a continuous function. Moreover from **(H4)**-1.(b), for almost every $(x_1, \ldots, x_n) \in \{(y_1, \ldots, y_n) \in Dom(f_1);\ 0 \leq y_1 + \ldots + y_n < x\}$:

$$\prod_{i=1}^{n} f_1(x_i, \alpha) \leq \prod_{i=1}^{n} \overline{f_1}(x_i)$$

where

$$(x_1, \ldots, x_n) \mapsto \prod_{i=1}^{n} \overline{f_1}(x_i)$$

is an integrable function on the domain $\{(y_1, \ldots, y_n) \in Dom(f_1);\ 0 \leq y_1 + \ldots + y_n < x\}$. Finally, due to the dominated convergence theorem (applied to the continuity of parametrized integrals), we deduce that $\alpha \mapsto \mathbb{P}[\sum_{i=0}^{n} X_i < x]$ is continuous. That proves that assumption A2 from [29] is verified. Thus, we can apply [29, Theorem 2.1] and the statement of Theorem 5.1 is proved.
□ In order to prove Theorem 5.2, we need the following lemma.

Lemma 2. Assume **(H5)** is verified and that for some $N \in \mathbb{N}$, $\alpha^* \in \Theta_1$, we have

$$\mathbb{P}_{\alpha^*}[X_1 + \ldots + X_N < x] = \mathbb{P}_{\alpha^*}[X_1 + \ldots + X_{N+1} < x].$$

Then, $\forall \alpha \in \Theta_1$, $\mathbb{P}_\alpha[X_1 + \ldots + X_N < x] = \mathbb{P}_\alpha[X_1 + \ldots + X_{N+1} < x]$.

Proof of Lemma 2. Assume that for some $N \in \mathbb{N}$, $\alpha^* \in \Theta_1$, we have

$$\mathbb{P}_{\alpha^*}[X_1 + \ldots + X_N < x] = \mathbb{P}_{\alpha^*}[X_1 + \ldots + X_{N+1} < x].$$

From lemma 1, this quantity is zero or one. If the lemma assumption is verified, then for some α^*, $Supp\{X_1 + \ldots + X_N\} \subset [x, +\infty[$ and consequently, $Supp\{X_1\} \subset [x/N, +\infty[$. Since the support of X_1 does not depend on α^*, then the same property is verified for any $\alpha \in \Theta_1$ and we obtain

$$0 \leq \mathbb{P}_\alpha[X_1 + \ldots + X_{N+1} < x] \leq \mathbb{P}_\alpha[X_1 + \ldots + X_N < x] = 0.$$

If $\mathbb{P}_{\alpha^*}[X_1 + \ldots + X_N < x] = 1$, as in the proof of Lemma 1, we define $M_{\max} = \sup\{t \in Supp\{X_1\}\}$; note that $M_{\max}$ does not depend on α^*. Once again from the proof of Lemma 1, we have $M_{\max} \leq x$ and that $Supp\{X_1 + \ldots + X_N\} \cap [0, x[\subset [0, x - M_{\max}[$.

Now let $\alpha \in \Theta_1$. Since $Supp\{X_1 + \ldots + X_N\} \cap [0, x[\subset [0, x - M_{\max}[$, and the support of X_1 does not depend on the parameter. We have

$$\begin{aligned} &\mathbb{P}_\alpha[X_1 + \ldots + X_N < x] \\ &= \mathbb{P}_\alpha[X_1 + \ldots + X_N < x - M_{\max}]\mathbb{P}_\alpha[X_1 \leq M_{\max}] \\ &= \mathbb{P}_\alpha[X_1 + \ldots + X_N < x - M_{\max}; X_{N+1} < M_{\max}] \leq \mathbb{P}_\alpha[X_1 + \ldots + X_{N+1} < x]. \end{aligned}$$

So $\mathbb{P}_\alpha[X_1 + \ldots + X_N < x] = \mathbb{P}_\alpha[X_1 + \ldots + X_{N+1} < x]$, and the proof is done.

Notations. In order to enlighten the formulas in the next theorem, we define

$$\begin{aligned} &\lambda_{\min} = \min\{\lambda \in \Lambda\}, \qquad \lambda_m = \max\{\lambda \in \Lambda\}, \\ &m_{n,X} = \min\{c_{n,X}(\alpha), \quad \alpha \in \Theta_1\}, \quad \widetilde{m}_{n,X} = \min\{c_{n,X}(\alpha) - c_{n+1,X}(\alpha), \quad \alpha \in \Theta_1\}, \\ &m_{n,Z} = \min\{c_{n,Z}(\beta), \quad \beta \in \Theta_2\}, \quad \widetilde{m}_{n,Z} = \min\{c_{n,Z}(\beta) - c_{n+1,Z}(\beta), \quad \beta \in \Theta_2\}. \end{aligned}$$

Under Conditions **(H3)** (compactness) and **(H4)** (regularity), these quantities are finite and non-negative.

Proof of Theorem 5.2.. In the proof of this theorem, we will only discuss on the case where we consider the censoring couple (Y, Δ) from Model I. The proof for the censoring couple (Y, Δ) from Model II uses the exact same arguments since the density of the model has a similar structure.
To prove this theorem, we need to check Conditions A1 to A3 of [29]. A1 and A2 have already been proved in the proof of Theorem 5.1.

We firstly prove that there exist N_1, N_2, N_3 in $\mathbb{N}$ such that $\widetilde{m}_{N_1,X} m_{N_1,Z} > 0$, $\widetilde{m}_{N_2,Z} m_{N_2+1,X} > 0$ and if $f_{(Y,\Delta)}(\,.\,,0) \neq 0$, $\widetilde{m}_{N_3,X}\widetilde{m}_{N_3,Z} > 0$. To prove it, we use the fact that $\mathbb{P}\left[T = C\right]$ is positive. Hence, we deduce from the formula of Lemma 3.2 the existence of $N_1 \in \mathbb{N}$ such that

$$(c_{N_1,X}(\alpha^0) - c_{N_1+1,X}(\alpha^0))(c_{N_1,Z}(\beta^0) - c_{N_1+1,Z}(\beta^0)) > 0.$$

And by applying Lemma 2 using a reductio ad absurdum, we deduce that $\widetilde{m}_{N_1,X}\widetilde{m}_{N_1,Z} > 0$. Since

$$(c_{n,X}(\alpha^0) - c_{n+1,X}(\alpha^0))(c_{n,Z}(\beta^0) - c_{n+1,Z}(\beta^0)) \leq (c_{n,X}(\alpha^0) - c_{n+1,X}(\alpha^0))c_{n,Z}(\beta^0),$$

and since the support of Z_1 does not depend on β, a similar proof justifies that $\widetilde{m}_{N_1,X} m_{N_1,Z} > 0$. To prove the third inequality $\widetilde{m}_{N_2,Z} m_{N_2+1,X} > 0$ for some $N_2 \in \mathbb{N}$ when $f_{(Y,\Delta)}(\,.\,,0) \neq 0$, assume ad absurdum that it is not the case. We can prove from Lemma 2 and from the fact that the support of X_1 does not depend on α that

$$f_{(Y,\Delta),\theta}(t,0) = 0 \qquad \forall t \geq 0,\ \forall \theta \in \Theta.$$

Hence $T \leq C$ a.s., which is also absurd.
Assumption A3. Uniqueness of the minimum of the Kullback-Leibler information criterion (hypothesis A3.b) is guaranteed here, by our parametric setting and our identifiability condition.
To have Condition A3.a of [29], we need to obtain a function that uniformly bounds the family of density functions $f_{(Y,\Delta),\theta}$ on $\Lambda \times \Theta_1 \times \Theta_2$, independently of θ. It is now easy to see that, if $\delta = 1$, for any $\theta \in \Lambda \times \Theta_1 \times \Theta_2$ and any $t > 0$,

$$e^{-\lambda_m t} \sum_{n \geq 0} \widetilde{m}_{n,X} m_{n,Z} \frac{(\lambda_{\min} t)^n}{n!} \leq f_{(Y,\Delta),\theta}(t,1) \leq e^{-\lambda_{\min} t} e^{\lambda_m t}.$$

Since there exists $N_1 \in \mathbb{N}$ such that $\widetilde{m}_{N_1,X} m_{N_1,Z} > 0$, then for any $t > 0$,

$$e^{-\lambda_m t} \widetilde{m}_{N_1,X} m_{N_1,Z} \frac{(\lambda_{\min} t)^{N_1}}{N_1!} \leq f_{(Y,\Delta),\theta}(t,1) \leq e^{(\lambda_m - \lambda_{\min})t}.$$

and thus for any $t > 0$

$$\begin{aligned} & |\log f_{(Y,\Delta),\theta}(t,1)| \\ \leq\ & \max\left\{ |\lambda_m - \lambda_{\min}| t; \left| -\lambda_m t + \log\left(\frac{\widetilde{m}_{N_1,X} m_{N_1,Z}}{N_1!}\right) N_1 \log(\lambda_{\min} t) \right| \right\}. \end{aligned}$$

The bounding function is independent of $\theta \in \Lambda \times \Theta_1 \times \Theta_2$ and is of linear growth w.r.t. t. Hence it is integrable with respect to $dF_{(Y,\Delta),(\lambda^0,\alpha^0,\beta^0)}$, the density function with true parameter $(\lambda^0, \alpha^0, \beta^0)$. Similarly the fact that there exists N_2 such that $\widetilde{m}_{N_2,Z} m_{N_2+1,X} > 0$ ensures the existence of a similar dominating function when $\delta = 0$ and when $f_{(Y,\Delta)}(\, . \, , 0) \neq 0$.

Since in our setting the function g in [29] is equal to $f_{(Y,\Delta),(\lambda^0,\alpha^0,\beta^0)}$, from the previous bounds, it follows that $\mathbb{E}[\log(g(Y,\Delta)]$ exists. This achieves the proof of Theorem 5.2. □

The following lemma will help to obtain proper uniform dominations of the parametric derivatives of the density functions in the proof of theorem 5.3.

Lemma 3. Assume that **(H3)**, **(H4)** and **(H5)** are verified. Then there exist $C_1 > 1$ and $N_1 \in \mathbb{N}^*$ such that

$$\forall n \geq N_1, \forall \alpha \in \Theta_1, \quad C_1 \mathbb{P}_\alpha[X_1 + \ldots + X_{n+1} < x] \leq \mathbb{P}_\alpha[X_1 + \ldots + X_n < x].$$

The same property holds for the sequence $(Z_n)_{n\in\mathbb{N}}$.

Proof of Lemma 3. We prove the result on the sequence $(X_n)_{n\in\mathbb{N}^*}$; the proof is the same for $(Z_n)_{n\in\mathbb{N}^*}$. Assumption **(H3)** guarantees that Θ_1 is a compact subset of $\mathbb{R}^{d_1}$ and assumption **(H5)** guarantees that the support of X_1 does not depend on α. We now need to distinguish whether $(X_n)_{n\in\mathbb{N}^*}$ belongs to the family F_1, F_2 or F_3 (see Definition 4.4). If $(X_n)_{n\in\mathbb{N}^*} \in F_1$, then $c_{n,X}(\alpha) = 0$ after some threshold N_1, which does not depend on α thanks to **(H5)**. Any constant $C_1 > 1$ does the job.
If $(X_n)_{n\in\mathbb{N}^*} \in F_2$, let $N_1(\alpha) = \min\{n \geq 2, c_{n-1,X}(\alpha) < 1\}$. Note that under assumptions **(H3)** and **(H4)**, there exists $N_1 \geq 2$ such that for all $\alpha \in \Theta_1$ and $n \geq N_1$, $c_{n,X}(\alpha) < 1$. Furthermore if $c_{N_1,X}(\alpha) = 0$, then for all $n \geq N_1$, $c_{n,X}(\alpha) = 0$ and any $C_1 > 1$ is suitable. Now we suppose that $0 < c_{N_1,X}(\alpha) < 1$. Then for all $n \geq N_1$ and $\alpha \in \Theta_1$

$$\begin{aligned}
&\frac{\mathbb{P}_\alpha[X_1 + \ldots + X_{n+1} < x]}{\mathbb{P}_\alpha[X_1 + \ldots + X_n < x]} \\
&= \frac{\int_0^x \phi_{S_{n+1-N_1},\alpha}(x-t)\mathbb{P}_\alpha[X_1 + \ldots + X_{N_1} < t]dt}{\int_0^x \phi_{S_{n+1-N_1},\alpha}(x-t)\mathbb{P}_\alpha[X_1 + \ldots + X_{N_1-1} < t]dt} \\
&= \frac{\int_0^x \phi_{S_{n+1-N_1},\alpha}(x-t)\mathbb{P}_\alpha[X_1 + \ldots + X_{N_1-1} < t]\frac{\mathbb{P}_\alpha[X_1+\ldots+X_{N_1}<t]}{\mathbb{P}_\alpha[X_1+\ldots+X_{N_1-1}<t]}dt}{\int_0^x \phi_{S_{n+1-N_1},\alpha}(x-t)\mathbb{P}_\alpha[X_1 + \ldots + X_{N_1-1} < t]dt}.
\end{aligned}$$

The function $h_{N_1} : (t,\alpha) \mapsto \frac{\mathbb{P}_\alpha[X_1+\ldots+X_{N_1}<t]}{\mathbb{P}_\alpha[X_1+\ldots+X_{N_1-1}<t]}$ is continuous on $]0,x]\times\Theta_1$ and for any $\alpha \in \Theta_1$, $\lim_{t\to 0} h_{N_1}(t,\alpha) = 0$. Thus, the function can be extended to a continuous function on the compact subset $[0,x]\times\Theta_1$, which is still denoted by h_{N_1}.
We have for any fixed $\alpha \in \Theta_1$, $\forall t \in [0,x]$, $h_{N_1}(t) < 1$. If it is not the case, for some $t \in]0,x]$, it follows that $\mathbb{P}_\alpha[X_1+\ldots+X_{N_1} < t] = \mathbb{P}_\alpha[X_1+\ldots+X_{N_1-1} < t]$ and Lemma 1 gives that $\mathbb{P}_\alpha[X_1+\ldots+X_{N_1} < t] \in \{0;1\}$. Since X_n is in F_2, it cannot be zero. If it is one, then $c_{N_1,X}(\alpha) = 1$, which contradicts our hypothesis. Thus h_{N_1} is a continuous function strictly dominated by 1 on $[0,x]\times\Theta_1$. Hence we deduce that there exists $K_1 < 1$ such that, $h_{N_1}(t,\alpha) \le K_1$, $\forall (t,\alpha) \in [0,x]\times\Theta_1$. Consequently

$$\frac{\mathbb{P}_\alpha[X_1+\ldots+X_{n+1}<x]}{\mathbb{P}_\alpha[X_1+\ldots+X_n<x]} \le K_1$$

and $C_1 = 1/K_1$ is a right constant.
If $(X_n)_{n\in\mathbb{N}^*} \in F_3$, in proof of Theorem 4.6, one can see that we obtained

$$\left|\frac{\mathbb{P}_\alpha[S_{n+1}<x]}{\mathbb{P}_\alpha[S_n<x]} - \mathbb{P}_\alpha[X_1=0]\right| \le \frac{C_x}{n}, \qquad \forall n \ge M_{\max,x}$$

where $C_x = (M_{\max,x-x_{\min}}+1)\frac{\mathbb{P}_\alpha[X_1=0]}{\mathbb{P}_\alpha[X_1=x_{\min}]}$, with $M_{\max,x}$ and $M_{\max,x-x_{\min}}$ that do not depend on α. Therefore

$$\frac{\mathbb{P}_\alpha[S_{n+1}<x]}{\mathbb{P}_\alpha[S_n<x]} \le (M_{\max,x-x_{\min}}+1)\frac{\mathbb{P}_\alpha[X_1=0]}{\mathbb{P}_\alpha[X_1=x_{\min}]}\frac{1}{n}\mathbb{P}_\alpha[X_1=0], \qquad \forall n \ge M_{\max,x}$$

where $\alpha \mapsto \frac{\mathbb{P}_\alpha[X_1=0]}{\mathbb{P}_\alpha[X_1=x_{\min}]}$ and $\alpha \mapsto \mathbb{P}_\alpha[X_1=0]$ are continuous functions on the compact subset Θ_1. We can consider $C_1^{-1} = \sup_{\alpha\in\Theta_1}\{(\mathbb{P}_\alpha[X_1=0]+1)/2\}$ as a uniform dominating constant.

Assumption 3 (Condition **(H6)**)**.** With the notations of Condition **(H4)** (see Assumption 2):

1. For all $u \in Dom(f_1)\cap[0,x]$ the function $\alpha \mapsto f_1(u,\alpha)$ is twice differentiable on $\overset{\circ}{\Theta}_1$ with:
   (a) $\forall \alpha \in \overset{\circ}{\Theta}_1$, $u \in]0,x] \mapsto \partial_\alpha f_1(u,\alpha)$ is measurable.
   (b) For any $i_1, i_2 \in \{1,\ldots,d_1\}$, there exists g_{1,i_1} and h_{1,i_1,i_2} two integrable functions with respect to ν_1 on $[0,x]$ such that:

$$\left|\frac{\partial f_1(u,\alpha)}{\partial\alpha_{i_1}}\right| \le g_{1,i_1}(u) \qquad \text{and} \qquad \left|\frac{\partial^2 f_1(u,\alpha)}{\partial\alpha_{i_1}\partial\alpha_{i_2}}\right| \le h_{1,i_1,i_2}(u) \qquad \nu_1 - a.e.$$

2. For all $u \in Dom(f_2) \cap [0, z]$, the function $\beta \mapsto f_2(u, \beta)$ is two times differentiable on $\overset{\circ}{\Theta}_2$ with:
   (a) $\forall \beta \in \overset{\circ}{\Theta}_2$, $u \in]0, z] \mapsto \partial_\beta f_2(u, \beta)$ is measurable.
   (b) For any $j_1, j_2 \in \{1, \ldots, d_2\}$, there exists g_{2,j_1} and h_{2,j_1,j_2} two integrable functions with respect to ν_2 on $[0, z]$ such that

$$\left|\frac{\partial f_2(u,\beta)}{\partial \beta_{j_1}}\right| \leq g_{2,j_1}(u) \qquad \text{and} \qquad \left|\frac{\partial^2 f_2(u,\beta)}{\partial \beta_{j_1}\partial \beta_{j_2}}\right| \leq h_{2,j_1,j_2}(u) \qquad \nu_2 - a.e.$$

Proof of Theorem 5.3.. In the proof of this theorem, we only discuss the case where we consider the censoring couple (Y, Δ) for Model I. The proof for the strict censoring couple for Model II uses the exact same arguments since the density of the second couple has a similar structure.

To prove this theorem, we need to verify assumption A1-A6 in [29]. The assumptions A1-A3 have already been proved in the previous proofs, so only the analysis of A4-A6 are made.

Assumption A4. Let us recall (Lemma 3.4) that, for any $(\lambda, \alpha, \beta) \in \Lambda \times \Theta_1 \times \Theta_2, t > 0$,

$$f_{(Y,\Delta),(\lambda,\alpha,\beta)}(t, 1) = \sum_{n=0}^{+\infty}[c_{n,X}(\alpha) - c_{n+1,X}(\alpha)]c_{n,Z}(\beta)\lambda e^{-\lambda t}\frac{(\lambda t)^n}{n!}$$
$$f_{(Y,\Delta),(\lambda,\alpha,\beta)}(t, 0) = \sum_{n=0}^{+\infty}[c_{n,Z}(\beta) - c_{n+1,Z}(\beta)]c_{n+1,X}(\alpha)\lambda e^{-\lambda t}\frac{(\lambda t)^n}{n!}$$

It easily comes from the previous assumption that A4 is verified in our case. In fact, if $\delta = 1$, then for all $t > 0$, $i \in \{1, \ldots, d_1\}$ and $j \in \{1, \ldots, d_2\}$,

$$\log(f_{(Y,\Delta),(\lambda,\alpha,\beta)}(t, 1)) = -\lambda t + \log\left(\sum_{n=0}^{+\infty}[c_{n,X}(\alpha) - c_{n+1,X}(\alpha)]c_{n,Z}(\beta)\lambda\frac{(\lambda t)^n}{n!}\right).$$

From the proof of Theorem 5.2, the term in the logarithm is positive and we

can compute all the derivatives. We have

$$\frac{\partial \log(f_{(Y,\Delta),(\lambda,\alpha,\beta)}(t,1))}{\partial \lambda} = -t + \frac{\sum_{n=0}^{+\infty}[c_{n,X}(\alpha) - c_{n+1,X}(\alpha)]c_{n,Z}(\beta)(n+1)\frac{(\lambda t)^n}{n!}}{\sum_{n=0}^{+\infty}[c_{n,X}(\alpha) - c_{n+1,X}(\alpha)]c_{n,Z}(\beta)\lambda\frac{(\lambda t)^n}{n!}} \tag{16}$$

$$\frac{\partial \log(f_{(Y,\Delta),(\lambda,\alpha,\beta)}(t,1))}{\partial \alpha_i} = \frac{\sum_{n=0}^{+\infty}\partial_{\alpha_i}[c_{n,X}(\alpha) - c_{n+1,X}(\alpha)]c_{n,Z}(\beta)\frac{(\lambda t)^n}{n!}}{\sum_{n=0}^{+\infty}[c_{n,X}(\alpha) - c_{n+1,X}(\alpha)]c_{n,Z}(\beta)\frac{(\lambda t)^n}{n!}} \tag{17}$$

$$\frac{\partial \log(f_{(Y,\Delta),(\lambda,\alpha,\beta)}(t,1))}{\partial \beta_j} = \frac{\sum_{n=0}^{+\infty}[c_{n,X}(\alpha) - c_{n+1,X}(\alpha)]\partial_{\beta_j}c_{n,Z}(\beta)\frac{(\lambda t)^n}{n!}}{\sum_{n=0}^{+\infty}[c_{n,X}(\alpha) - c_{n+1,X}(\alpha)]c_{n,Z}(\beta)\frac{(\lambda t)^n}{n!}}.$$

The same computations hold when $\delta = 0$. Since for any $(\lambda,\alpha,\beta) \in \Lambda \times \Theta_1 \times \Theta_2$, all of these above functions are continuous in t, whether $\delta = 0$ or $\delta = 1$ on $(\mathbb{R}_+^*, \mathcal{B}(\mathbb{R}_+^*))$, it is clear that they are measurable in $(t,\delta) \in \mathbb{R}_+^* \times \{0,1\}$. The fact that functions are continuously differentiable comes from all the assumptions made earlier and from theorem of derivation of parametrized integrals.

Assumption A5. We need to get uniform bounds for the first and second derivatives w.r.t. the parameters $\theta = (\lambda,\alpha,\beta) \in \Lambda \times \Theta_1 \times \Theta_2$.

Let start with the first derivative w.r.t. λ. For $\delta = 1$, with (16), we have

$$\frac{\partial \log(f_{(Y,\Delta),(\lambda,\alpha,\beta)}(t,1))}{\partial \lambda} = -t + \frac{1}{\lambda} + t\frac{\sum_{n=0}^{+\infty}[c_{n+1,X}(\alpha) - c_{n+2,X}(\alpha)]c_{n+1,Z}(\beta)\frac{(\lambda t)^n}{n!}}{\sum_{n=0}^{+\infty}[c_{n,X}(\alpha) - c_{n+1,X}(\alpha)]c_{n,Z}(\beta)\frac{(\lambda t)^n}{n!}}.$$

From Lemma 3, it follows that for all $n \geq N_1$ and $\alpha \in \Theta_1$,

$$0 \leq [c_{n+1,X}(\alpha) - c_{n+2,X}(\alpha)] \leq \frac{1}{C_1 - 1}[c_{n,X}(\alpha) - c_{n+1,X}(\alpha)].$$

And

$$\begin{aligned}
&\sum_{n=0}^{N_1}[c_{n+1,X}(\alpha) - c_{n+2,X}(\alpha)]c_{n+1,Z}(\beta)\frac{(\lambda t)^n}{n!} \\
&= \frac{1}{\lambda t}\sum_{m=1}^{N_1+1}[c_{m,X}(\alpha) - c_{m+1,X}(\alpha)]c_{m,Z}(\beta)m\frac{(\lambda t)^m}{m!} \\
&\leq \frac{N_1+1}{\lambda t}\sum_{m=1}^{N_1+1}[c_{m,X}(\alpha) - c_{m+1,X}(\alpha)]c_{m,Z}(\beta)\frac{(\lambda t)^m}{m!}.
\end{aligned}$$

Hence we immediately obtain the following uniform dominating function: for $t > 0$,

$$\left|\frac{\partial \log(f_{(Y,\Delta),(\lambda,\alpha,\beta)}(t,1))}{\partial \lambda}\right| \leq t + \frac{1}{\lambda_{\min}} + \frac{N_1+1}{\lambda_{\min}} + \frac{t}{C_1-1} = m_1(t)$$

For $\delta = 0$, similar reflections ensures the following uniform dominating function: for $t > 0$,

$$\left|\frac{\partial \log(f_{(Y,\Delta),(\lambda,\alpha,\beta)}(t,0))}{\partial \lambda}\right| \leq |t| + \frac{1}{\lambda_{\min}} + \frac{N_2+1}{\lambda_{\min}} + \frac{|t|}{C_2-1} = m_2(t)$$

where we used Lemma 3 to have the existence of $C_2 > 1$ and $N_2 \in \mathbb{N}^*$ such that $\forall n \geq N_2$ and $\forall \beta \in \Theta_2$, $C_2\mathbb{P}_\beta[Z_1+\ldots+Z_{n+1} < z] \leq \mathbb{P}_\beta[Z_1+\ldots+Z_n < z]$. Now we want to control the derivative

$$(t,\delta) \mapsto \frac{\partial \log(f_{(Y,\Delta),(\lambda,\alpha,\beta)}(t,\delta))}{\partial \alpha_{i_1}}, \quad i \in \{1,\ldots,d_1\},$$

given by (17) for $\delta = 1$. To obtain a convenient upper bound, we need to study the following terms,

$$\partial_{\alpha_i}[c_{n,X}(\alpha) - c_{n+1,X}(\alpha)] \qquad \text{and} \qquad \partial_{\alpha_i} c_{n,X}(\alpha)$$

for n sufficiently large. As explained at the end of the verification of the assumption A4, those terms are well defined due to the theorem of derivation of parametrized integrals and moreover with **(H6)**

$$\begin{aligned}
\partial_{\alpha_i} c_{n,X}(\alpha) &= n\int_{\mathbb{R}_+^n} \mathbb{1}_{\{x_1+\ldots+x_n<x\}} \prod_{k=1}^{n-1} f_1(x_k,\alpha)\partial_{\alpha_i} f_1(x_n,\alpha) d\nu_1(x_1)\ldots d\nu_1(x_n) \\
&= n\int_0^x \mathbb{P}_\alpha[X_1+\ldots+X_{n-1} < x - x_n]\partial_{\alpha_i} f_1(x_n,\alpha) d\nu_1(x_n),
\end{aligned}$$

thus

$$\begin{aligned}
|\partial_{\alpha_i} c_{n,X}(\alpha)| &\leq n\mathbb{P}_\alpha[X_1+\ldots+X_{n-1} < x] \int_0^x |\partial_{\alpha_i} f(x_n,\alpha)| d\nu_1(x_n) \\
&\leq n c_{n-1,X}(\alpha) \int_0^x g_{1,i}(x_n) d\nu_1(x_n),
\end{aligned}$$

where $\int_0^x g_{1,i}(x_n)d\nu_1(x_n)$ is finite. By the same way,

$$|\partial_{\alpha_i}[c_{n,X}(\alpha)-c_{n+1,X}(\alpha)]| \leq \left[nc_{n-1,X}(\alpha)+(n+1)c_{n,X}(\alpha)\right]\int_0^x g_{1,i}(x_n)d\nu_1(x_n).$$

Pose $\bar{g}_{1,i} = \int_0^x g_{1,i}(x_n)d\nu_1(x_n)$ and $M_{1,\min,\alpha} = \min\{n \in \mathbb{N}; \quad c_{n,X}(\alpha) - c_{n+1,X}(\alpha) > 0\}$, $\alpha \in \Theta_1$. Combining Lemma 1 and 2, $M_{1,\min,\alpha} = M_{1,\min}$ does not depend on α, and for any $n \in \{0;\ldots;M_{1,\min}-1\}$,

$$c_{n,X}(\alpha) - c_{n+1,X}(\alpha) = \partial_{\alpha_i}\left(c_{n,X}(\alpha) - c_{n+1,X}(\alpha)\right) = 0.$$

Then using (17), we deduce

$$\begin{aligned}
\left|\frac{\partial \log(f_{(Y,\Delta),(\lambda,\alpha,\beta)}(t,1))}{\partial\alpha_i}\right| &\leq \bar{g}_{1,i}\frac{\sum_{n=M_{1,\min}}^{+\infty} n[c_{n-1,X}(\alpha)+c_{n,X}(\alpha)]c_{n,Z}(\beta)\frac{(\lambda t)^n}{n!}}{\sum_{n=M_{1,\min}}^{+\infty}[c_{n,X}(\alpha)-c_{n+1,X}(\alpha)]c_{n,Z}(\beta)\frac{(\lambda t)^n}{n!}} \\
&+ \bar{g}_{1,i}\frac{\sum_{n=M_{1,\min}}^{+\infty} c_{n,X}(\alpha)c_{n,Z}(\beta)\frac{(\lambda t)^n}{n!}}{\sum_{n=M_{1,\min}}^{+\infty}[c_{n,X}(\alpha)-c_{n+1,X}(\alpha)]c_{n,Z}(\beta)\frac{(\lambda t)^n}{n!}} \\
&\leq \bar{g}_{1,i}\lambda t\frac{\sum_{n=M_{1,\min}}^{+\infty}[c_{n,X}(\alpha)+c_{n+1,X}(\alpha)]c_{n+1,Z}(\beta)\frac{(\lambda t)^n}{n!}}{\sum_{n=M_{1,\min}}^{+\infty}[c_{n,X}(\alpha)-c_{n+1,X}(\alpha)]c_{n,Z}(\beta)\frac{(\lambda t)^n}{n!}} \\
&+ \bar{g}_{1,i}\frac{\sum_{n=M_{1,\min}}^{+\infty} c_{n,X}(\alpha)c_{n,Z}(\beta)\frac{(\lambda t)^n}{n!}}{\sum_{n=M_{1,\min}}^{+\infty}[c_{n,X}(\alpha)-c_{n+1,X}(\alpha)]c_{n,Z}(\beta)\frac{(\lambda t)^n}{n!}}.
\end{aligned}$$

And with Lemma 3 for any $n \geq N_1$, $\alpha \in \Theta_1$,

$$\begin{aligned}
[c_{n,X}(\alpha)+c_{n+1,X}(\alpha)] &\leq \frac{C_1+1}{C_1-1}[c_{n,X}(\alpha)-c_{n+1,X}(\alpha)] \\
c_{n,X}(\alpha) &\leq \frac{C_1+1}{C_1-1}[c_{n,X}(\alpha)-c_{n+1,X}(\alpha)].
\end{aligned}$$

Hence

$$\begin{aligned}
&\frac{\sum_{n=M_{1,\min}}^{+\infty}[c_{n,X}(\alpha)+c_{n+1,X}(\alpha)]c_{n+1,Z}(\beta)\frac{(\lambda t)^n}{n!}}{\sum_{n=M_{1,\min}}^{+\infty}[c_{n,X}(\alpha)-c_{n+1,X}(\alpha)]c_{n,Z}(\beta)\frac{(\lambda t)^n}{n!}} \\
&\quad\leq \frac{\sum_{n=M_{1,\min}}^{N_1}[c_{n,X}(\alpha)+c_{n+1,X}(\alpha)]c_{n+1,Z}(\beta)\frac{(\lambda t)^n}{n!}}{\sum_{n=M_{1,\min}}^{+\infty}[c_{n,X}(\alpha)-c_{n+1,X}(\alpha)]c_{n,Z}(\beta)\frac{(\lambda t)^n}{n!}} + \frac{C_1+1}{C_1-1} \\
&\quad\leq \frac{\sum_{n=M_{1,\min}}^{N_1}[c_{n,X}(\alpha)+c_{n+1,X}(\alpha)]c_{n+1,Z}(\beta)\frac{(\lambda t)^n}{n!}}{[c_{M_{1,\min},X}(\alpha)-c_{M_{1,\min}+1,X}(\alpha)]c_{M_{1,\min},Z}(\beta)\frac{(\lambda t)^{M_{1,\min}}}{M_{1,\min}!}} + \frac{C_1+1}{C_1-1}.
\end{aligned}$$

From Lemma 3.2, since we work in a censoring model, for all $\beta \in \Theta_2$, $c_{M_{1,\min},Z}(\beta) > 0$. Consequently, we obtain the following domination,

$$\left|\frac{\partial \log(f_{(Y,\Delta),(\lambda,\alpha,\beta)}(t,1))}{\partial \alpha_i}\right| \leq \bar{g}_{1,i}\frac{C_1+1}{C_1-1}(1+\lambda_m t) + \frac{2\bar{g}_{1,i}(1+\lambda_m t)}{\tilde{m}_{M_{1,\min},X} m_{M_{1,\min},Z}} \sum_{n=0}^{N_1-M_{1,\min}} (\lambda_m t)^n.$$

When $\delta = 0$,

$$\begin{aligned}
&\left|\frac{\partial \log(f_{(Y,\Delta),(\lambda,\alpha,\beta)}(t,0))}{\partial \alpha_i}\right| \\
&\leq \bar{g}_{1,i}\frac{\sum_{n=0}^{+\infty}[c_{n,Z}(\beta)-c_{n+1,Z}(\beta)](n+1)c_{n,X}(\alpha)\frac{(\lambda t)^n}{n!}}{\sum_{n=0}^{+\infty}[c_{n,Z}(\beta)-c_{n+1,Z}(\beta)]c_{n+1,X}(\alpha)\frac{(\lambda t)^n}{n!}} \\
&= \bar{g}_{1,i}\frac{(1-c_{1,Z}(\beta)) + (\lambda t)\sum_{n=0}^{+\infty}[c_{n+1,Z}(\beta)-c_{n+2,Z}(\beta)]\frac{n+2}{n+1}c_{n+1,X}(\alpha)\frac{(\lambda t)^n}{n!}}{\sum_{n=0}^{+\infty}[c_{n,Z}(\beta)-c_{n+1,Z}(\beta)]c_{n+1,X}(\alpha)\frac{(\lambda t)^n}{n!}} \\
&\leq \bar{g}_{1,i}\frac{(1-c_{1,Z}(\beta)) + 2\lambda t\sum_{n=0}^{+\infty}[c_{n+1,Z}(\beta)-c_{n+2,Z}(\beta)]c_{n+1,X}(\alpha)\frac{(\lambda t)^n}{n!}}{\sum_{n=0}^{+\infty}[c_{n,Z}(\beta)-c_{n+1,Z}(\beta)]c_{n+1,X}(\alpha)\frac{(\lambda t)^n}{n!}}.
\end{aligned}$$

If $1 - c_{1,Z}(\beta) > 0$, then

$$\frac{(1-c_{1,Z}(\beta))}{\sum_{n=0}^{+\infty}[c_{n,Z}(\beta)-c_{n+1,Z}(\beta)]c_{n+1,X}(\alpha)\frac{(\lambda t)^n}{n!}} \leq \frac{1}{c_{1,X}(\alpha)} \leq \frac{1}{m_{1,X}}.$$

Indeed if $c_{1,X}(\alpha) = 0$, $X_1 \geq x$ a.s. which implies that $T \leq C$ a.s., which is excluded. Hence if $M_{2,\min} = M_{2,\min,\beta} = \min\{n \in \mathbb{N};\ \ c_{n,Z}(\beta) - c_{n+1,Z}(\beta) > 0\}$, we obtain

$$\begin{aligned}
&\left|\frac{\partial \log(f_{(Y,\Delta),(\lambda,\alpha,\beta)}(t,0))}{\partial \alpha_i}\right| \\
&\leq \bar{g}_{1,i}\frac{1}{m_{1,X}} + 2\bar{g}_{1,i}\lambda t\frac{\sum_{n=N_{2,\min}-1}^{+\infty}[c_{n+1,Z}(\beta)-c_{n+2,Z}(\beta)]c_{n+1,X}(\alpha)\frac{(\lambda t)^n}{n!}}{\sum_{n=M_{2,\min}}^{+\infty}[c_{n,Z}(\beta)-c_{n+1,Z}(\beta)]c_{n+1,X}(\alpha)\frac{(\lambda t)^n}{n!}}.
\end{aligned}$$

As in the case $\delta = 1$, we separate the sums in two parts $n \leq N_2$ and $n \geq N_2$, where N_2 is given by Lemma 3. For $n \geq N_2$, we have a bound equal to

$2\bar{g}_{1,i}\lambda t \dfrac{1}{C_2 - 1}$. For $M_{2,\min} \leq n \leq N_2$, we obtain a polynomial bound as in the case $\delta = 1$. Thus we obtain a polynomial bound

$$\left| \frac{\partial \log(f_{(Y,\Delta),(\lambda,\alpha,\beta)}(t,0))}{\partial \alpha_i} \right|$$
$$\leq \bar{g}_{1,i} \frac{1}{m_{1,X}} + 2\bar{g}_{1,i} \frac{1}{\tilde{m}_{M_{2,\min},Z} m_{M_{2,\min},X}} \sum_{n=0}^{N_2 - M_{2,\min}} (\lambda_m t)^n + \frac{2\bar{g}_{1,i}(\lambda_m t)}{C_2 - 1}.$$

Hence in both cases $\delta = 1$ or $\delta = 0$, we have a polynomial bound on the derivative. And any polynomial function is integrable with respect to $F_{(Y,\Delta),(\lambda^0,\alpha^0,\beta^0)}$. The exact same reflections allow us to obtain the same type of inequalities for all of the remaining derivatives of first order

$$(t, \delta) \mapsto \frac{\partial \log(f_{(Y,\Delta),(\lambda,\alpha,\beta)}(t,\delta))}{\partial \beta_{j_1}},$$

with $j_1 \in \{1, \ldots, d_2\}$ and of second order

$$\frac{\partial^2 \log(f_{(Y,\Delta),(\lambda,\alpha,\beta)}(t,\delta))}{\partial \lambda^2}, \quad \frac{\partial^2 \log(f_{(Y,\Delta),(\lambda,\alpha,\beta)}(t,\delta))}{\partial \alpha_{i_1} \partial \lambda}, \quad \frac{\partial^2 \log(f_{(Y,\Delta),(\lambda,\alpha,\beta)}(t,\delta))}{\partial \beta_{j_1} \partial \lambda}$$
$$\frac{\partial^2 \log(f_{(Y,\Delta),(\lambda,\alpha,\beta)}(t,\delta))}{\partial \alpha_{i_1} \partial \alpha_{i_2}}, \quad \frac{\partial^2 \log(f_{(Y,\Delta),(\lambda,\alpha,\beta)}(t,\delta))}{\partial \beta_{j_1} \alpha_{i_2}}, \quad \frac{\partial^2 \log(f_{(Y,\Delta),(\lambda,\alpha,\beta)}(t,\delta))}{\partial \beta_{j_1} \partial \beta_{j_2}}$$

for any $i_1, i_2 \in \{1, \ldots, d_1\}$, $j_1, j_2 \in \{1, \ldots, d_2\}$. There are polynomial functions that respectively dominate all these derivatives, uniformly on the family of parameters $(\lambda, \alpha, \beta) \in \Lambda \times \Theta_1 \times \Theta_2$, which proves that A5 holds.

Assumption A6. We already assume that $(\lambda^0, \alpha^0, \beta^0) \in \mathring{\Theta}$ in **(H3)**. Evoke that the matrices $A(\theta)$ and $B(\theta)$ are defined by Equations (8) and (9). Furthermore since the model is correctly specified, $A(\theta) = -B(\theta)$; we prove this property below (also see remark after [29, Theorem 3.2]). We also assume that $A(\theta^0) = -B(\theta^0)$ is non-singular. Therefore Assumption A6 of [29] holds.

The conclusion of Theorem 5.3 follows now from [29, Theorem 3.2]. □

Lemma 4. For all $\theta \in \mathring{\Theta}$, $A(\theta) = -B(\theta)$.

Proof of Lemma 4. We only prove the result considering (Y, Δ) from Model I, since the proofs with Model II are the same. To prove it, we can compute the terms of both matrices and we show that for all $i, j \in \{1, \ldots, 1+d_1+d_2\}$

$$\mathbb{E}[\partial^2 \log(f_{(Y,\Delta),\theta}/\partial\theta_i\partial\theta_j] = -\mathbb{E}[\big(\partial \log(f_{(Y,\Delta),\theta}/\partial\theta_j\big)\ \big(\partial \log(f_{(Y,\Delta),\theta}/\partial\theta_i\big)].$$

Indeed, if $(\theta_i, \theta_j) = (\lambda, \lambda)$,

$$\begin{aligned}
&\mathbb{E}\left[\frac{\partial^2 \log(f_{(Y,\Delta),(\lambda,\alpha,\beta)})}{\partial\lambda^2}\right] \\
&= \int_0^{+\infty} \frac{\partial^2 \log(f_{(\lambda,\alpha,\beta)}(y,1))}{\partial\lambda^2} f_{(\lambda,\alpha,\beta)}(y,1)dy \\
&\quad + \int_0^{+\infty} \frac{\partial^2 \log(f_{(\lambda,\alpha,\beta)}(y,0))}{\partial\lambda^2} f_{(\lambda,\alpha,\beta)}(y,0)dy \\
&= \int_0^{+\infty} \frac{\partial^2 f_{(\lambda,\alpha,\beta)}(y,1)}{\partial\lambda^2}dy - \int_0^{+\infty} \frac{(\partial_\lambda f_{(\lambda,\alpha,\beta)}(y,1))^2}{f_{(\lambda,\alpha,\beta)}(y,1)}dy \\
&\quad + \int_0^{+\infty} \frac{\partial^2 f_{(\lambda,\alpha,\beta)}(y,0)}{\partial\lambda^2}dy - \int_0^{+\infty} \frac{(\partial_\lambda f_{(\lambda,\alpha,\beta)}(y,0))^2}{f_{(\lambda,\alpha,\beta)}(y,0)}dy
\end{aligned}$$

and

$$\begin{aligned}
&\mathbb{E}\left[\left(\frac{\partial \log(f_{(\lambda,\alpha,\beta)}(Y,\Delta))}{\partial\lambda}\right)^2\right] \\
&= \int_0^{+\infty} \frac{(\partial_\lambda f_{(\lambda,\alpha,\beta)}(y,1))^2}{f_{(\lambda,\alpha,\beta)}(y,1)}dy + \int_0^{+\infty} \frac{(\partial_\lambda f_{(\lambda,\alpha,\beta)}(y,0))^2}{f_{(\lambda,\alpha,\beta)}(y,0)}dy.
\end{aligned}$$

Now

$$\begin{aligned}
&\int_0^{+\infty} \frac{\partial^2 f_{(\lambda,\alpha,\beta)}(y,1)}{\partial\lambda^2}dy \\
&= \int_0^{+\infty} \partial_\lambda^2 \sum_{n\geq 0}(c_{n,X}(\alpha) - c_{n+1,X}(\alpha))c_{n,Z}(\beta)e^{-\lambda y}\lambda\frac{(\lambda y)^n}{n!}dy \\
&\quad = \int_0^{+\infty} \sum_{n\geq 0}(c_{n,X}(\alpha) - c_{n+1,X}(\alpha))c_{n,Z}(\beta)e^{-\lambda y}\left[(n+1)n - 2(n+1)y\lambda + y^2\lambda^2\right]\lambda^{n-1}\frac{y^n}{n!}dy \\
&\quad = \sum_{n\geq 0}(c_{n,X}(\alpha) - c_{n+1,X}(\alpha))c_{n,Z}(\beta)\left[n - 2(n+1) + (n+2)\right]\frac{n+1}{\lambda^2} = 0,
\end{aligned}$$

where we used that for all $n \in \mathbb{N}$

$$\int_0^{+\infty} e^{-\lambda y} \lambda^{n+1} \frac{(\lambda y)^n}{n!} dy = 1$$

and the inversion of integral and series, which is valid thanks to the convergence theorem 4.6. Since the structure of $y \mapsto f_{(\lambda,\alpha,\beta)}(y,1)$ and $y \mapsto f_{(\lambda,\alpha,\beta)}(y,0)$ is the same, we also obtain,

$$\int_0^{+\infty} \frac{\partial^2 f_{(\lambda,\alpha,\beta)}(y,0)}{\partial \lambda^2} dy = 0.$$

Similar arguments show that

$$\int_0^{+\infty} \frac{\partial^2 f_{(\lambda,\alpha,\beta)}(y,1)}{\partial \lambda \partial \theta_j} dy = \int_0^{+\infty} \frac{\partial^2 f_{(\lambda,\alpha,\beta)}(y,0)}{\partial \lambda \partial \theta_j} dy = 0$$

if $\theta_j = \alpha_k$, $1 \leq k \leq d_1$ or $\theta_j = \beta_k$, $1 \leq k \leq d_2$. Indeed,

$$\begin{aligned}
&\int_0^{+\infty} \frac{\partial^2 f_{(\lambda,\alpha,\beta)}(y,1)}{\partial \lambda \partial \alpha_k} dy \\
&= \int_0^{+\infty} \partial_\lambda \partial_{\alpha_k} \sum_{n \geq 0} (c_{n,X}(\alpha) - c_{n+1,X}(\alpha)) c_{n,Z}(\beta) e^{-\lambda y} \lambda \frac{(\lambda y)^n}{n!} dy \\
&= \int_0^{+\infty} \sum_{n \geq 0} \partial_{\alpha_k} (c_{n,X}(\alpha) - c_{n+1,X}(\alpha)) c_{n,Z}(\beta) \left[(n+1) - y\lambda\right] e^{-\lambda y} \lambda^n \frac{y^n}{n!} dy \\
&= \sum_{n \geq 0} \partial_{\alpha_k} (c_{n,X}(\alpha) - c_{n+1,X}(\alpha)) c_{n,Z}(\beta) \left[(n+1) - (n+1)\right] \frac{1}{\lambda} = 0.
\end{aligned}$$

Finally if $(\theta_i, \theta_j) = (\alpha_k, \beta_l)$ for some $k \in \{1, \ldots, d_1\}$, $l \in \{1, \ldots, d_2\}$, we have

$$\begin{aligned}
&\int_0^{+\infty} \frac{\partial^2 f_{(\lambda,\alpha,\beta)}(y,1)}{\partial \alpha_k \partial \beta_l} dy \\
&= \int_0^{+\infty} \partial_{\alpha_k} \partial_{\beta_l} \sum_{n \geq 0} (c_{n,X}(\alpha) - c_{n+1,X}(\alpha)) c_{n,Z}(\beta) e^{-\lambda y} \lambda \frac{(\lambda y)^n}{n!} dy \\
&= \partial_{\alpha_k} \partial_{\beta_l} \sum_{n \geq 0} (c_{n,X}(\alpha) - c_{n+1,X}(\alpha)) c_{n,Z}(\beta) \int_0^{+\infty} e^{-\lambda y} \lambda \frac{(\lambda y)^n}{n!} dy \\
&= \partial_{\alpha_k} \partial_{\beta_l} \sum_{n \geq 0} (c_{n,X}(\alpha) - c_{n+1,X}(\alpha)) c_{n,Z}(\beta)
\end{aligned}$$

and

$$\int_0^{+\infty} \frac{\partial^2 f_{(\lambda,\alpha,\beta)}(y,0)}{\partial\alpha_k \partial\beta_l} dy$$
$$= \int_0^{+\infty} \partial_{\alpha_k}\partial_{\beta_l} \sum_{n\geq 0} (c_{n,Z}(\beta) - c_{n+1,Z}(\beta)) c_{n+1,X}(\alpha) e^{-\lambda y} \lambda \frac{(\lambda y)^n}{n!} dy$$
$$= \partial_{\alpha_k}\partial_{\beta_l} \sum_{n\geq 0} (c_{n,Z}(\beta) - c_{n+1,Z}(\beta)) c_{n+1,X}(\alpha) \int_0^{+\infty} e^{-\lambda y} \lambda \frac{(\lambda y)^n}{n!} dy$$
$$= \partial_{\alpha_k}\partial_{\beta_l} \sum_{n\geq 0} (c_{n,Z}(\beta) - c_{n+1,Z}(\beta)) c_{n+1,X}(\alpha).$$

Thus,

$$\int_0^{+\infty} \frac{\partial^2 f_{(\lambda,\alpha,\beta)}(y,1)}{\partial\alpha_k \partial\beta_l} dy + \int_0^{+\infty} \frac{\partial^2 f_{(\lambda,\alpha,\beta)}(y,0)}{\partial\alpha_k \partial\beta_l} dy$$
$$= \partial_{\alpha_k}\partial_{\beta_l} \sum_{n\geq 0} (c_{n,X}(\alpha) - c_{n+1,X}(\alpha)) c_{n,Z}(\beta) + (c_{n,Z}(\beta) - c_{n+1,Z}(\beta)) c_{n+1,X}(\alpha)$$
$$= \partial_{\alpha_k}\partial_{\beta_l} \sum_{n\geq 0} c_{n,X}(\alpha) c_{n,Z}(\beta) - c_{n+1,Z}(\beta) c_{n+1,X}(\alpha)$$
$$= \partial_{\alpha_k}\partial_{\beta_l} c_{0,X}(\alpha) c_{0,Z}(\beta) = 0,$$

since $\alpha \mapsto c_{0,X}(\alpha)$ and $\beta \mapsto c_{0,Z}(\beta)$ are constant functions. We use the same arguments if $(\theta_i, \theta_j) = (\alpha_k, \alpha_l)$ or if $(\theta_i, \theta_j) = (\beta_k, \beta_l)$, which achieves the proof.
